# Divergent Adsorption Regulation in Metal–Organic Frameworks for Highly Efficient CF_4_/C_2_F_6_ Separation

**DOI:** 10.1002/advs.202411083

**Published:** 2024-12-04

**Authors:** Guihong Xu, Tian Ke, Rongrong Fan, Kaiyuan Tan, Wenjun Zhang, Baogen Su, Zhiguo Zhang, Zongbi Bao, Qilong Ren, Qiwei Yang

**Affiliations:** ^1^ Key Laboratory of Biomass Chemical Engineering of Ministry of Education College of Chemical and Biological Engineering, Zhejiang University Hangzhou 310027 China; ^2^ Institute of Zhejiang University‐Quzhou Quzhou 324000 China

**Keywords:** adsorption, divergent regulation, gas separation, metal–organic frameworks, perfluorocarbons

## Abstract

The efficient removal of low‐concentration components from homologous mixtures is often hampered by the co‐directional effect of traditional thermodynamic regulation approaches, typically leading to a trade‐off between adsorption capacity and selectivity. Focusing this challenge on the critical task of purifying perfluorocarbons in electronics industry, a divergent regulation strategy is reported that significantly improves the separation efficiency of low‐concentration hexafluoroethane (C_2_F_6_) from tetrafluoromethane (CF_4_). This approach involves the selective shielding of open metal sites and the modulation of channel geometry within an electron‐deficient ligand‐based pore environment, thereby facilitating a C_2_F_6_ dense‐packing accommodation mode while weakening the CF_4_ affinity due to the reduced host‐guest interactions. Simultaneously enhanced C_2_F_6_ adsorption and reduced CF_4_ adsorption are achieved, resulting in record‐high low‐pressure C_2_F_6_ uptake and C_2_F_6_/CF_4_ selectivity. Comprehensive insights into the unique separation mechanism are illustrated through a combination of solid‐state MAS nuclear magnetic resonance (SSNMR), molecular simulations, and meticulously designed comparative experiments. As a result, benchmark C_2_F_6_/CF_4_ separation performance is achieved, as demonstrated by the unprecedented electronic‐grade (over 99.999%) CF_4_ productivity (401 L kg^−1^) obtained from an industrially relevant C_2_F_6_/CF_4_ (3:97) mixture, as well as the excellent water/air/heat stability and recyclability.

## Graphical abstract

By avoiding overly strong host‐guest interactions and instead introducing pore environments that selectively promote larger‐size C_2_F_6_ to stack more tightly, Zn(BPZ) achieves a ideal scenario in which C_2_F_6_ adsorption increases while CF_4_ adsorption decreases, enhances capacity and selectivity simultaneously, obtains high‐purity CF_4_ with high productivity and yield.

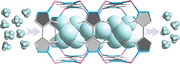

## 1 Introduction

Electronic specialty gases are essential to the semiconductor manufacturing industry, often regarded as the “blood” of this sector. The global market for these gases was ≈$5 billion in 2022, with projection exceeding $6 billion by 2025.^[^ ^1^ ^]^ Among these, fluorinated gases (F‐gases) account for roughly 30% of the total usage, with tetrafluoromethane (CF_4_) being one of the most in‐demand, primarily utilized in processes such as semiconductor etching and cleaning.^[^ ^2^, ^3^ ^]^ Conventional CF_4_ production is typically accompanied by other perfluorocarbons (PFCs) such as hexafluoroethane (C_2_F_6_), tetrafluoroethylene (C_2_F_4_), and octafluoropropane (C_3_F_8_). These impurities must be deeply removed to obtain electronic‐grade (>99.999%) CF_4_ for high‐end applications. However, the efficient separation of these similar F‐gases presents significant challenges, particularly for the separation of C_2_F_6_ (0.01–3%),^[^ ^4^, ^5^, ^6^ ^]^ which has the most striking similarity in physical and molecular properties to CF_4_ among the PFCs.

Cryogenic distillation remains the primary method used for C_2_F_6_/CF_4_ separation. However, the challenge posed by the low boiling points of C_2_F_6_ (−78.2 °C) and CF_4_ (−128 °C), coupled with the low concentration of C_2_F_6_, result in high energy consumption and low efficiency.^[^ ^2^, ^7^, ^8^ ^]^ In comparison, physisorption using porous solid adsorbents has emerged as an energy‐saving, promising alternative to distillation due to the absence of a phase change, especially for separating low‐concentration components.^[^ ^9^, ^10^, ^11^, ^12^, ^13^ ^]^ The efficiency of this method largely depends on the properties of the adsorbent. Nevertheless, traditional porous adsorbents, such as activated carbon and zeolite 13X, exhibit restricted selectivity (6.3 and 8.6 at 303 K and 100 kPa, respectively) and adsorption capacity (0.24 and 0.21 mmol C_2_F_6_ g^−1^ at 303 K and 3 kPa, respectively).^[^ ^14^, ^15^ ^]^ Two fluorine‐functionalized porous organic frameworks (F‐POFs) named SPPOF‐4F and SPPOF‐8F have been reported for the separation of C_2_F_6_/CF_4_.^[^ ^16^ ^]^ But their selectivity (6.6 and 8.0 at 298 K and 100 kPa, respectively) and capacity (0.16 and 0.15 mmol C_2_F_6_ g^−1^ at 298 K and 3 kPa, respectively) are unsatisfactory. This limitation is due to the nonpolar, saturated nature of both C_2_F_6_ and CF_4_ molecules, which have zero dipole moments, similar kinetic diameters (5.1 Å for C_2_F_6_ and 4.7 Å for CF_4_), and low polarizabilities (68.2 × 10^−25^ cm^3^ for C_2_F_6_ and 38.4 × 10^−25^ cm^3^ for CF_4_).

Metal‐organic frameworks (MOFs), a burgeoning class of porous materials self‐assembled from metal nodes and organic ligands, have received significant attention in the field of gas capture and separation. Their appeal lies in the remarkable diversity of pore structures together with the on‐demand tunable surface properties.^[^ ^17^, ^18^ ^]^ Over the past twenty years, MOFs have demonstrated their efficiency in a range of applications, such as CO_2_ capture, light hydrocarbon separations, and fluorinated gases recovery from N_2_.^[^ ^19^, ^20^ ^]^ However, despite these advancements, there is still a considerable gap in the research focused on the separation of C_2_F_6_/CF_4_ using MOFs, especially given the extremely similar structure and properties of CF_4_ and C_2_F_6_ as described above. It remains an uncertain issue whether high selectivity and high adsorption capacity can be achieved to obtain electronic‐grade CF_4_.

**Scheme 1:**
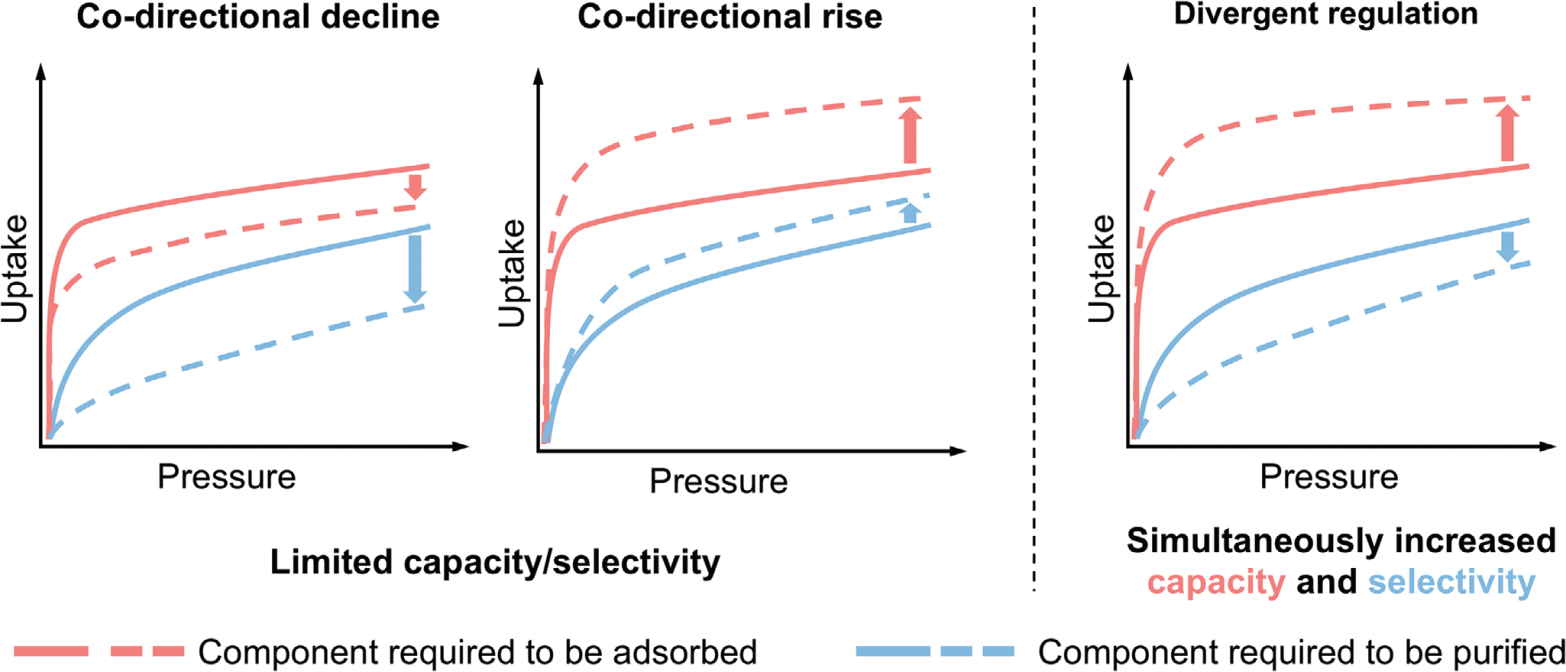
Comparison of adsorption isotherm changes under different modes of pore regulation.

Size sieving is frequently heralded as an ideal mechanism for achieving high selectivity by selectively excluding the larger‐size molecule from mixtures via appropriately sized apertures.^[^ ^21^, ^22^ ^]^ Unfortunately, this approach proves inefficient for separating low concentration larger‐size molecules (e.g., C_2_F_6_ in CF_4_), where an excessive increase in adsorbent dosage is necessary to adsorb the predominant CF_4_ in feed gas while excluding small amounts of C_2_F_6_, and extra regeneration process is required to obtain pure CF_4_. Similarly, kinetic separation based on molecular size discrepancies is also unsuitable in such contexts. Therefore, a more pragmatic approach involves reducing the affinity of high‐concentration CF_4_ while enhancing the adsorption of larger‐size C_2_F_6_ on adsorbent, within the thermodynamic mechanism. Nonetheless, conventional pore‐tuning strategies for thermodynamically driven adsorbents—such as the incorporation of strong binding sites or the regulation of pore size—often lead to co‐directional regulation, wherein the adsorption of both components either increases or decreases concurrently^[^ ^23^, ^24^, ^25^ ^]^ (**Scheme** **1**). This phenomenon is particularly problematic when separating homologous molecules with highly close thermodynamic properties, such as C_2_H_6_/CH_4_,^[^ ^10^, ^26^ ^]^ thereby hindering the concurrent enhancement of adsorption capacity and selectivity.

Herein, we reported a unique adsorption divergent regulation strategy that effectively addresses the challenging separation of dilute C_2_F_6_ from CF_4_. By avoiding overly strong host‐guest interactions and instead introducing pore environments that selectively promote larger‐size C_2_F_6_ to stack more tightly, the ideal scenario in which C_2_F_6_ adsorption increases while CF_4_ adsorption decreases can be achieved. Among the two electron‐deficient ligand‐based MOFs studied, Ni(BPZ) (BPZ = 4,4′‐Bi‐pyrazole) with Lewis acidic open metal sites (OMSs) showed strong affinity for both C_2_F_6_ and CF_4_, both having predominantly negative electrostatic potential (ESP). In contrast, Zn(BPZ), despite comparable specific surface area but with blocked metal sites, exhibited higher C_2_F_6_ adsorption and lower CF_4_ adsorption than Ni(BPZ), nearly doubling the C_2_F_6_/CF_4_ selectivity. A combination of density functional theory with dispersion corrections (DFT‐D), grand canonical Monte Carlo (GCMC) simulations and SSNMR spectra revealed that the phenomenon is driven by weaker host‐guest interactions in Zn(BPZ), coupled with favorable C_2_F_6_ tight‐stacking behavior, owing to differences in the pore structure/binding mode compared to Ni(BPZ). Record low‐pressure C_2_F_6_ uptake and C_2_F_6_/CF_4_ selectivity of Zn(BPZ), together with its outstanding stability against water, air, and heat, position it as one of the most promising candidates for CF_4_ purification. Fixed‐bed breakthrough experiments further confirmed that Ni(BPZ) and Zn(BPZ) can achieve >99.999% CF_4_ purity, with Zn(BPZ) yielding an exceptional electronic‐grade CF_4_ productivity up to 401 L kg^−1^—four times that of Ni(BPZ) and demonstrated excellent recycling performance.

## 2 Results and Discussion

**Figure 1:**
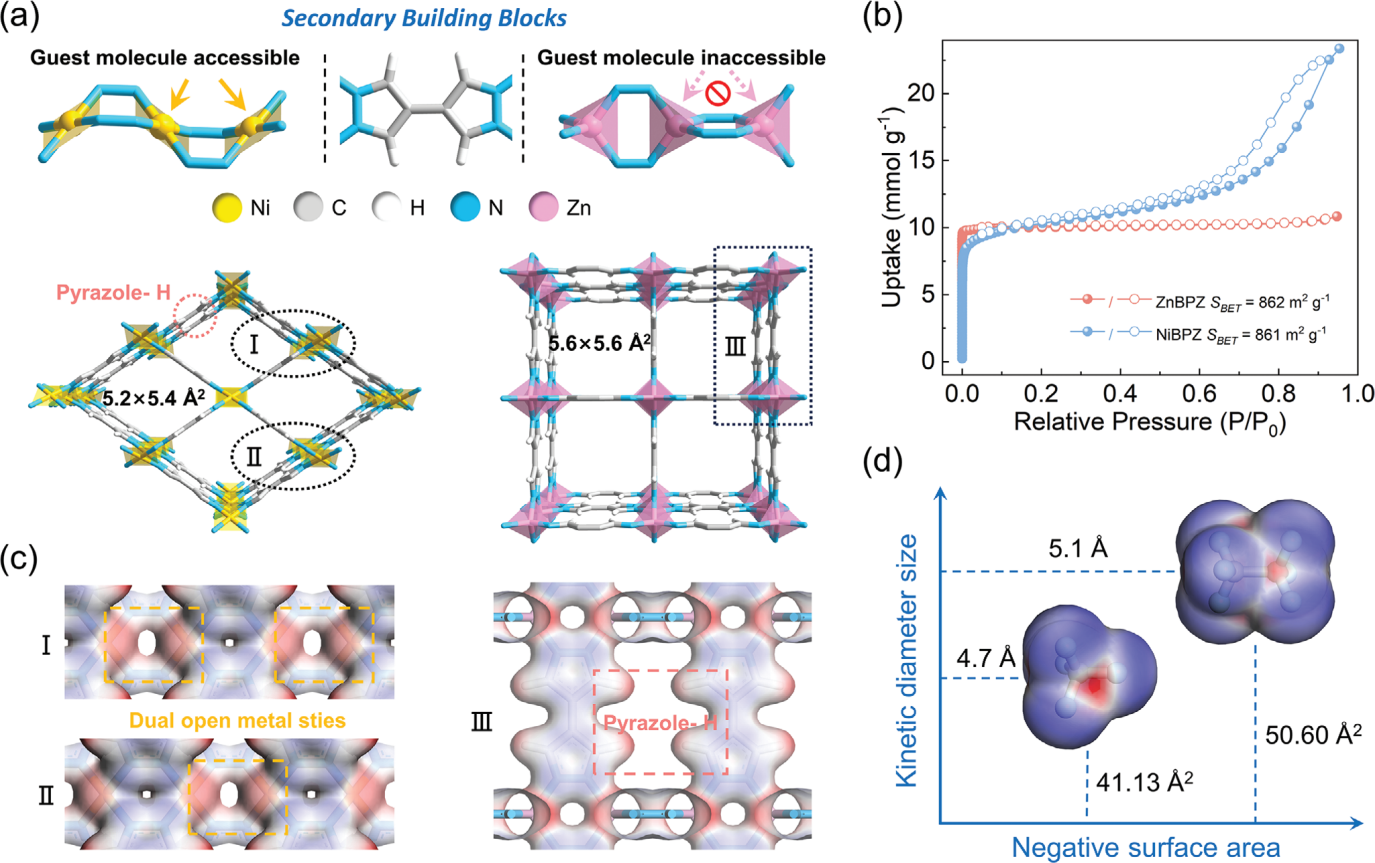
The demonstration of the structure of Ni(BPZ), Zn(BPZ), CF_4_ and C_2_F_6_. a) The secondary building blocks and crystal structure of Ni(BPZ) and Zn(BPZ). b) The N_2_ adsorption isotherms of Ni(BPZ) and Zn(BPZ) at 77K. c) The ESP surface of Ni(BPZ) and Zn(BPZ) with a scale spanning from −0.05 (blue) to 0.20 a.u. (red) and a transparency of 10%. d) The ESP surface of CF_4_ and C_2_F_6_ with a scale spanning from −0.01 (blue) to 0.04 a.u. (red) and a transparency of 20%.

Ni(BPZ) and Zn(BPZ) can be obtained simply by heating and stirring in the presence of deprotonating reagents according to the previous report.^[^ ^27^ ^]^ As shown in **Figure** **1**a, Ni(BPZ) crystallizes in the orthorhombic space group *Imma*, where Ni(II) are coordinated by the N atoms in the four independent BPZ ligands and form a square planar structure, which can form OMSs accessible to guest molecules when the solvent molecules are removed. The NiN_4_ planes with two orientations distribute along the b‐axis alternately, where N atoms bridge successive metal nodes. The pore structure of Ni(BPZ) adopts a 1D rhombic shape, with a static pore size of 5.2 × 5.4 Å^2^. In contrast, Zn(BPZ) crystallizes in the tetragonal space group *P*4_2_/*mmc*, with two orientations and alternating ZnN_4_ tetrahedra distributed along the c‐axis direction. The pore structure of Zn(BPZ) adopts a 1D square shape, with a static pore size of 5.6 × 5.6 Å^2^. From the perspective of atomic orbital analysis, the d‐orbital configuration of Ni(II) is 3d^8^ (not fully occupied), and the splitting of d‐orbitals in planar square field lead to an empty d‐orbital in Ni(II) according to the Hund's rule, which tends to form a square planar coordination in dsp^2^ hybridization when it is coordinated with N atoms from the strong field pyrazole‐based ligands. Meanwhile, there is an empty 4p‐orbital remaining in Ni(II) after coordinating with bipyrazole, suggesting the ability to accept electrons as OMSs. However, the d‐orbital configuration of Zn(II) is 3d^10^ (fully occupied), tends not to be involved in the metal‐linker bonding due to its high energy.^[^ ^28^ ^]^ Consequently, Zn(II) exhibits a lack of OMSs and a square pore structure is formed when coordinates with bipyrazole by sp^3^ hybridization. The surface ESP distribution reveals that the accessible pore surface of both materials is mostly positive, particularly in the vicinity of the H atoms on the pyrazole (Figure 1c). Notably, in the framework of Ni(BPZ), each pair of adjacent Ni atoms is automatically positioned at the obtuse angles of the rhombic pores, creating an accessible dual open metal sites where the surface ESP is mainly positive (Figure 1a,c). In contrast, the tetrahedrally coordinated Zn atoms in Zn(BPZ) are encircled by nitrogen atoms, with much less guest accessible positive ESP region coverage. This phenomenon implies a relatively weaker host‐guest affinity of Zn(BPZ) compared to Ni(BPZ) for CF_4_ and C_2_F_6_ whose surface is mainly negative (Figure 1d).

The powder X‐ray diffraction (PXRD) of as‐synthesized Zn(BPZ) and Ni(BPZ) were in agreement with simulated patterns of their crystal structure (Figures S1 and S2, Supporting Information), which proved the bulk purity of them. Furthermore, both two materials retained their crystallinity upon exposure to air or immersion in water for months. The thermogravimetric analysis (Figure S10, Supporting Information) delineates the excellent thermal stability of Zn(BPZ) and Ni(BPZ), with their decomposition temperatures surpassing 723 and 623 K, respectively. To characterize the permanent porosity of the synthesized materials, the adsorption of N_2_ on the activated samples was determined at 77 K, as shown in Figure 1b. Ni(BPZ) exhibited a secondary rise in the N_2_ isotherm and a hysteresis loop of type H4, which may be attributed to the mesopore formed by particle stacking or defects.^[^ ^29^, ^30^ ^]^ The specific surface areas of Zn(BPZ) and Ni(BPZ), calculated using the Brunauer‐Emmett‐Teller (BET) method, were determined to be 861 and 862 m^2^ g^−1^, respectively (Figure S11, Supporting Information), implying the similar porosity of the two materials despite their different pore shape. The pore size distribution analysis, based on the non‐linear density functional theory (NLDFT) model, revealed the most probable pore diameters to be 5.6 Å for Zn(BPZ) and 5.4 Å for Ni(BPZ) (Figure S12, Supporting Information), in consistent with the results of the structural analysis. Water (H_2_O) adsorption isotherms at 298 K were performed to characterize the hydrophilicity of these two MOFs. As shown in Figure S19 (Supporting Information), the H_2_O uptake of Ni(BPZ) is higher than that of Zn(BPZ) at the same pressure. The stronger affinity of Ni(BPZ) for water was attributed to the OMSs.

**Figure 2:**
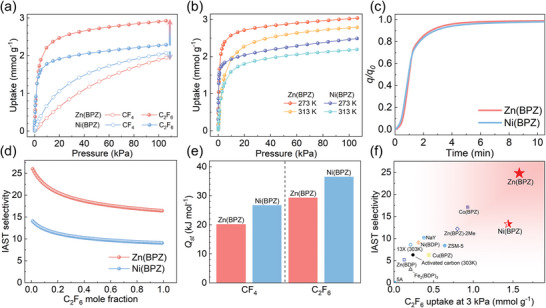
Gas adsorption separation properties of Ni(BPZ) and Zn(BPZ). a) The single‐component isotherms of C_2_F_6_ and CF_4_ on Ni(BPZ) and Zn(BPZ) at 298 K. b) The single‐component isotherms of C_2_F_6_ on Ni(BPZ) and Zn(BPZ) at 273 K and 313 K, respectively. c) Kinetic adsorption measurements of C_2_F_6_ in Ni(BPZ) and Zn(BPZ) at 3 kPa. d) The IAST selectivity of Ni(BPZ) and Zn(BPZ) to various ratios of C_2_F_6_/CF_4_ gas mixture (ranging from 1% to 99% C_2_F_6_) at 1 bar and 298 K. e) Isosteric heat of adsorption (*Q_st_*) at zero loading for C_2_F_6_ and CF_4_ of Ni(BPZ) and Zn(BPZ), respectively. f) Comparison of the C_2_F_6_ uptake at 3 kPa and the IAST selectivity of C_2_F_6_/CF_4_ mixture (3/97, v/v) on different adsorbents at 1 bar and 298 K.

The single‐component adsorption isotherms of CF_4_ and C_2_F_6_ on Zn(BPZ) and Ni(BPZ) were measured at 273 K, 298 K, and 313 K, respectively (**Figure** **2**a,b; Figure S20, Supporting Information). The results showed that both materials exhibited preferential adsorption of C_2_F_6_ over CF_4_, which is due to the molecular size of C_2_F_6_ being closer to the pore size of both materials than CF_4_. And as expected, the CF_4_ adsorption of Zn(BPZ), which has almost the same specific surface area as Ni(BPZ) and has no OMSs, was significantly lower than that of Ni(BPZ) throughout the entire interval from 0 to 100 kPa. However, to our surprise, Zn(BPZ) showed a higher C_2_F_6_ uptake (1.58 mmol g^−1^) at 298 K and 3 kPa than Ni(BPZ) (1.44 mmol g^−1^), and the uptake value further increased to 2.90 mmol g^−1^ at 100 kPa, indicating a substantial increase of 28% compared to that of Ni(BPZ) (2.27 mmol g^−1^). Furthermore, the temperature‐independent of the phenomenon indicates a consistent behavior under thermal variations (Figure 2b; Figure S20, Supporting Information). In other words, we achieved divergent adsorption regulation for CF_4_/C_2_F_6_ separation, where the uptake of the weakly adsorbed component decreases while that of the strongly adsorbed component increases from Ni(BPZ) to Zn(BPZ). This divergence implies that the adsorption mechanisms for C_2_F_6_ and CF_4_ within the two materials may be distinct. Kinetic adsorption experiments of C_2_F_6_ at 3 kPa in both Ni(BPZ) and Zn(BPZ) demonstrated that the adsorption of C_2_F_6_ by these materials is exceedingly rapid, as illustrated in Figure 2c. In addition, the kinetic adsorption isotherms of CF_4_ also showed no significant differences between the two materials (Figure S36, Supporting Information). Consequently, it is evident that the unexpected divergent changes in static adsorption are not attributable to kinetic constraints.

The adsorption selectivity on Ni(BPZ) and Zn(BPZ) was predicted using ideal adsorbed solution theory (IAST) at 298 K for C_2_F_6_/CF_4_ gas mixtures. As shown in Figure 2d, When the ratio of C_2_F_6_/CF_4_ is 3/97, the IAST selectivity for Ni(BPZ) was 13.3, surpassing that of conventional adsorbents, such as activated carbon (6.3, 303 K),^[^ ^14^ ^]^ zeolite 13X (8.6, 303K),^[^ ^14^ ^]^ ZSM‐5 (8.4) and NaY (10.2). The Zn(BPZ) with its higher C_2_F_6_ uptake and lower CF_4_ uptake, achieved an unprecedented selectivity of 24.8 for the C_2_F_6_/CF_4_ mixture, which is twice as high as Ni(BPZ). Moreover, the C_2_F_6_ adsorption capacity of Zn(BPZ) significantly exceeds the conventional adsorbents (Figure 2f; Table S5, Supporting Information). The combination of high C_2_F_6_/CF_4_ selectivity and high C_2_F_6_ capacity at low pressure renders Zn(BPZ) highly promising for separation applications.

The Isosteric heat of adsorption (*Q_st_*) of C_2_F_6_ and CF_4_ on Ni (BPZ) and Zn (BPZ) was determined based on the Virial‐type thermal equation. As shown in Figure 2e, at 298 K, the *Q_st_* values at zero loading for C_2_F_6_ and CF_4_ on Ni(BPZ) are 36.5 and 26.7 kJ mol^−1^, respectively. In contrast, the corresponding *Q_st_* values on Zn(BPZ) are 29.3 and 20.1 kJ mol^−1^, respectively, indicating its lower affinity for both two gases. This difference is attributed to the presence of positive OMSs in the structure of Ni(BPZ), which enhance the host‐guest interaction.

**Figure 3:**
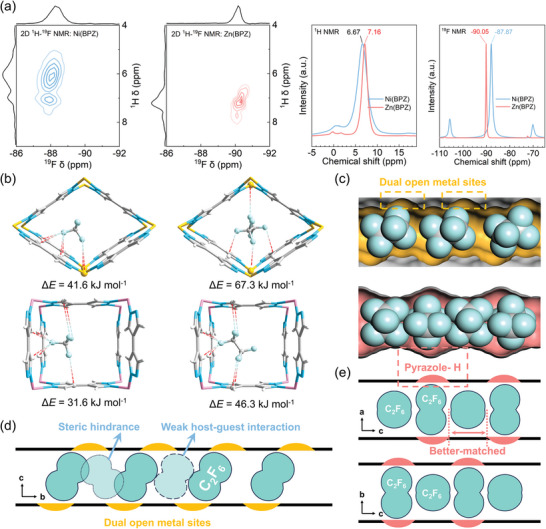
The molecular‐level adsorption mechanism of C_2_F_6_ and CF_4_ on Ni(BPZ) and Zn(BPZ), respectively. a) SSNMR spectra of C_2_F_6_ adsorbed on Ni(BPZ) and Zn(BPZ), respectively. b) Preferential binding sites simulated by DFT‐D for CF_4_ and C_2_F_6_ in Ni(BPZ) and Zn(BPZ), respectively. c) Snapshots of multi‐C_2_F_6_ loading simulation results in the channels of Ni(BPZ) and Zn(BPZ), respectively. Schematic diagram of the distribution of C_2_F_6_ at high loading in the channels of d) Ni(BPZ) and e) Zn(BPZ).

In order to further reveal the mechanism underlying the divergent adsorption change of C_2_F_6_ and CF_4_ by the two materials, SSNMR was conducted on Zn(BPZ) and Ni(BPZ) loaded with C_2_F_6_ at 1 bar to characterize the host‐guest interaction (**Figure** **3**a). In the 1D ^19^F spectrum, the corresponding chemical shift of F‐peak in Ni(BPZ) is larger than that in Zn(BPZ). It has been reported previously that the strong polarizing effect of F atoms modified on ligands can induce nonpolar guest molecular polarization to enhance dipolar interactions and adsorption perpormance (e.g., MIL‐101(Cr)‐4F (1%) for H_2_, Xe, and I_2_).^[^ ^31^ ^]^ The polariziation and dipolar interactions may exist between F atoms presented in C_2_F_6_ and frameworks, too. Correspondingly, the different ^19^F chemial shift in Ni(BPZ) and Zn(BPZ) implied that F atoms can also be affected by the positive confined environment, and a more pronounced effect on C_2_F_6_ was observed in Ni(BPZ). Meanwhile, the broader F‐peak in Ni(BPZ) indicated a relatively more fixed position of the C_2_F_6_ molecules within the channels.^[^ ^32^ ^]^ Moreover, the 2D ^1^H – ^19^F spectra revealed a F···H interaction between C_2_F_6_ and both materials.^[^ ^33^ ^]^

Based on the preliminary insight from the SSNMR, GCMC simulations were conducted to model the adsorption of guest molecules at various loading. Subsequently, the obtained loaded structures were optimized using DFT‐D,^[^ ^34^ ^]^ and the binding energies were calculated. The single‐loading computational results indicated that the adsorption of PFCs primarily relied on multiple van der Waals interactions with the positive regions within the frameworks (Figure 3b). Within the channels of Ni(BPZ), a fluorine atom from the CF_4_ molecule was captured by the pyrazole hydrogen (with F···H distances ranging from 2.81 to 3.00 Å), while another fluorine atom was trapped in the OMSs (with F···Ni distances of 3.81 Å). In comparison, for the larger C_2_F_6_ molecule, each perfluoromethyl group exhibited strong interactions with Ni(BPZ) similar to CF_4_ (with the F···H distances of 3.01–3.20 Å and F···Ni distances of 3.60–3.66 Å). Ni(BPZ) captured multiple parts of the molecule through multiple sites in the pore, which resulted in a fixed position of the absorbed C_2_F_6_ (consistent with the results of solid‐state NMR) and a strong host‐guest interaction. The calculated binding energies of Ni(BPZ). for CF_4_ and C_2_F_6_ were 41.6 and 67.3 kJ mol^−1^, respectively. For Zn(BPZ), it was observed that the framework can only capture one F atom from CF_4_ or C_2_F_6_ through the positive H atoms on adjacent pyrazole rings due to the absence of OMSs (with F···H distances of 2.64–2.79 Å for CF_4_ and F···H distances of 2.60–2.67 Å for C_2_F_6_, respectively). The calculated binding energies of CF_4_ and C_2_F_6_ on Zn(BPZ) were 31.6 and 46.3 kJ mol^−1^, respectively, which are notably lower than those of Ni(BPZ), in agreement with the *Q*_st_ results.

After gradually increasing the molecular loading of PFCs in the supercell, the calculated binding energies of PFCs with Ni(BPZ) were all significantly larger than those of Zn(BPZ) and hardly varied with the loading (Tables S6–S9, Supporting Information). Considering their similar structures and specific surface areas, it seems that the adsorption performance of Ni(BPZ) on CF_4_ and C_2_F_6_ should also be superior to that of Zn(BPZ) across the board, but the results of the C_2_F_6_ adsorption experiments present an actual adsorption situation that is not the case (Figure 2a,b).

The high C_2_F_6_ loading structure (Figure 3c) obtained by simulation showed that there are large gaps between C_2_F_6_ molecules absorbed in the channels of Ni(BPZ). C_2_F_6_ molecules can only be accommodated within the channels of Ni(BPZ) in a sequential, uniform orientation, because of the staggered arrangement of OMSs. Whereas within the channels of Zn(BPZ), the C_2_F_6_ molecules are in closer proximity, and the F atoms are favorably positioned near the positive C regions (Figure 1d) on neighboring guest molecules, resulting in stronger guest‐guest interactions (4–6 kJ mol^−1^, Tables S6–S9, Supporting Information). Furthermore, the GCMC simulations revealed a maximum loading of 26 C_2_F_6_ molecules in the supercell of [Zn(BPZ)]_40_, which is larger than that calculated in the supercell of [Ni(BPZ)]_40_ (21 C_2_F_6_ at most). Both of these are very close to the maximum loading converted from the saturation capacities of C_2_F_6_ fitted by the dual‐site Langmuir‐Freundlich (DSLF) model (27.6 for Zn(BPZ) and 22.9 for Ni(BPZ)).

Based on the above analysis, we can conclude that the reason leading to the higher C_2_F_6_ uptake of Zn(BPZ) than Ni(BPZ) over a wide range of pressures is that the distribution of interaction sites in Zn(BPZ) is more suitable for C_2_F_6_ stacking compared to Ni(BPZ), which means a more tightly stacking and a higher space utilization rate. Although Ni(BPZ) exhibits stronger interactions with C_2_F_6_, its interaction site distribution is not optimally aligned with the guest molecule. Consequently, other alternative adsorption locations are either obstructed by steric hindrance relative to the optimal locations or characterized by weak host‐guest interactions, as shown in Figure 3d. In the case of Zn(BPZ), the position within the pore channel of C_2_F_6_ molecules is mainly influenced by the region of H in pyrazole. The distribution of these regions is more compatible with the size of the C_2_F_6_ molecules, facilitating a tighter stacking arrangement in the channels (Figure 3e).

**Figure 4:**
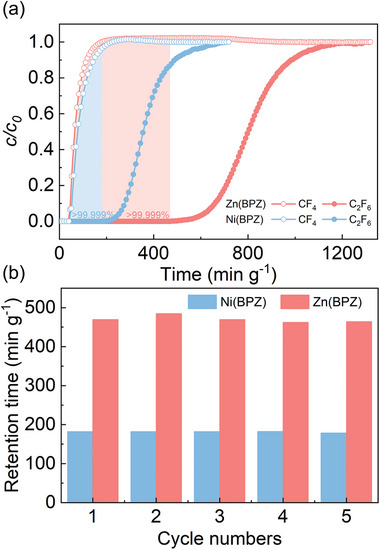
The practical separation of Ni(BPZ) and Zn(BPZ) for 3/97 C_2_F_6_/CF_4_ mixtures. a) Experimental breakthrough curves for C_2_F_6_/CF_4_ mixture (3/97, v/v) at 298 K on Ni(BPZ) and Zn(BPZ) columns, respectively. b) Retention time of C_2_F_6_ on the Ni(BPZ) and Zn(BPZ) columns with outlet concentration of CF_4_>99.999% in five dynamic cycles.

To further evaluate the practical separation of these two materials for 3/97 C_2_F_6_/CF_4_ mixtures, dynamic breakthrough experiments were performed at 298 K and 1 bar. As shown in **Figure** **4**a, when the mixed gas was introduced into the Ni(BPZ) column, CF_4_ penetrated the column first with a purity exceeding 99.999%, and the productivity of high‐purity (>99.999%) CF_4_ reached 94 L kg^−1^. The dynamic adsorption capacity of C_2_F_6_ was calculated to be 0.44 mmol g^−1^, and the dynamic selectivity of C_2_F_6_/CF_4_ was 7.4. Compared with Ni(BPZ), CF_4_ penetrated faster from the Zn(BPZ) column, with a dynamic capacity for C_2_F_6_ of 1.05 mmol g^−1^ and a dynamic selectivity of 26.3. The increased uptake of C_2_F_6_ and the decreased uptake of CF_4_ resulted in a significantly enhancement in the high‐purity CF_4_ productivity, reaching 401 L kg^−1^, which is 4‐times that of Ni(BPZ). Additionally, the yield of CF_4_ was improved (50.0% for Ni(BPZ) and 85.4% for Zn(BPZ), respectively). Zn(BPZ) achieved a simultaneous increase in both yield and productivity, indicating that the actual separation performance aligns well with the trends in adsorption capacity and selectivity predicted by static adsorption experiments. The adsorption‐saturated Ni(BPZ) and Zn(BPZ) can be readily regenerated through desorption by a helium purge at 333 K. Meanwhile, in five consecutive breakthrough cycles, Ni(BPZ) and Zn(BPZ) still maintained the retention time of high‐purity CF_4_, demonstrating their outstanding recyclability for C_2_F_6_/CF_4_ separation (Figure 4b). Additionally, the results of dynamic breakthrough experiments with C_2_F_6_/CF_4_ 1/99 gas mixture demonstrated that both materials can obtain high‐purity CF_4_ (≥99.999%), and the productivity reached 153 L kg^−1^ for Ni(BPZ) and 582 L kg^−1^ for Zn(BPZ) (Figure S43, Supporting Information). The dynamic adsorption capacity of C_2_F_6_ was calculated to be 0.17 mmol g^−1^ for Ni(BPZ) and 0.49 mmol g^−1^ for Zn(BPZ).

For the sake of completeness of the study and as a control experiment, we synthesized six MOFs by applying the ligand modification, ligand extension, and metal substitution strategies. And then tested their adsorption performances for CF_4_ and C_2_F_6_ at 298 K. Compared to Zn(BPZ), Zn(BPZ‐2Me)^[^ ^35^ ^]^ (BPZ‐2Me = 3,3′‐Dimethyl‐4,4′‐Bi‐pyrazole) showed a diminished adsorption capacity for both CF_4_ and C_2_F_6_ (Figure S24, Supporting Information). The reduction in adsorption is attributed to the decrease in pyrazole H due to methyl substitution, which lead to a weakening of the host‐guest interaction and a notable decline in the specific surface area. Zn(BDP) and Ni(BDP)^[^ ^36^ ^]^ (BDP = 1,4‐Di(‐pyrazol‐4‐yl)‐benzene) share the same topology with Zn(BPZ) and Ni(BPZ), respectively. However, both of them exhibited poor adsorption performance of C_2_F_6_ at low pressure (Figures S23 and S26, Supporting Information). This situation also occurred in Fe_2_(BDP)_3_^[^ ^37^ ^]^ with triangular channels (Figure S28, Supporting Information). The longer ligands will weaken the host‐guest interactions. Cu(BPZ)^[^ ^27^ ^]^ share the same topology with Ni(BPZ), yet its lower specific surface area results in a diminished adsorption capacity for PFCs (Figure S27, Supporting Information). This phenomenon may be attributed to the difficulty of completely removal of the solvent acetonitrile that are coordinated to the Cu^2+^. Co(BPZ)^[^ ^27^ ^]^ exhibited a co‐directional reduction in the adsorption of CF_4_ and C_2_F_6_ compared to Zn(BPZ) and Ni(BPZ) (Figure S25, Supporting Information). But unfortunately, the PXRD of Co(BPZ) was contaminated by fluorescence, which hampered its complete structural characterization. Given that the structure of Co(BDP)^[^ ^38^ ^]^ has already been reported and its topology aligns with that of Zn(BPZ), the structure of Zn(BPZ) was used as a template to substitute the metal ions with Co^2+^ and the structure was optimized using DFT‐D. The optimized structure of Co(BPZ) showed that the original square pore distorted. Considering its similar specific surface areas with Zn(BPZ) and Ni(BPZ), it is hypothesized that Co(BPZ) is neither able to have strong interactions for PFCs as Ni(BPZ) nor favorable for C_2_F_6_ stacking as Zn(BPZ).

## 3 Conclusion

In summary, this work reported a divergent adsorption regulation mechanism for the separation of CF_4_ and C_2_F_6_, a gas separation process of great importance to the electronics industry but challenging. Through the practice of Ni(BPZ) and Zn(BPZ) in the separation of C_2_F_6_/CF_4_, it is concluded that the key to achieve divergent regulation lies in avoiding overly strong host‐guest interactions and introducing pore environments that selectively promote larger‐size impurity moleculars to stack more tightly. An ideal scenario was achieved, with the selective enhancement of C_2_F_6_ adsorption while reducing CF_4_ uptake, despite their homologous nature. The combination of SSNMR, molecular simulations, and meticulously designed comparative experiments involving six materials revealed that the divergent modulation is rooted in the suitable strength and spatial distribution of binding sites of Zn(BPZ), which weakens the host‐guest interactions with CF_4_ and enhances the filling efficiency of C_2_F_6_ in the channels simultaneously. Consequently, Zn(BPZ) achieved exceptional productivity of high‐purity (over 99.999%) CF_4_ from a typical industrial mixture as well as excellent recyclability under the premise of high stability. Overall, this study not only reports an effective strategy for the adsorption separation of structurally similar PFCs, but also provides new inspiration for simultaneously improving the adsorption capacity and selectivity of adsorbents to solve the general challenge of efficiently separating low‐concentration but slightly larger molecules from gas product.

## Conflict of Interest

The authors declare no conflict of interest.

## Supporting information



Supporting Information

## Data Availability

The data that support the findings of this study are available from the corresponding author upon reasonable request.
