# Supplementary material for: Divergent Adsorption Regulation in Metal–Organic Frameworks for Highly Efficient CF4/C2F6 Separation
Source: Adv Sci (Weinh). 2024 Dec 4;12(4):2411083. doi: 10.1002/advs.202411083 (PMC11775566; doi:10.1002/advs.202411083)
Supplement: Supplementary file 1 — Supporting Information [file ADVS-12-2411083-s001.pdf]

ADVANCED SCIENCE
Open Access

# Supporting Information

for *Adv. Sci.*, DOI 10.1002/advs.202411083

Divergent Adsorption Regulation in Metal–Organic Frameworks for Highly Efficient $CF_4/C_2F_6$ Separation

*Guihong Xu, Tian Ke*, Rongrong Fan, Kaiyuan Tan, Wenjun Zhang, Baogen Su, Zhiguo Zhang, Zongbi Bao, Qilong Ren and Qiwei Yang**

Supporting Information
©Wiley-VCH 2024
69451 Weinheim, Germany

# Divergent adsorption regulation in metal-organic frameworks for highly efficient $CF_4/C_2F_6$ separation

Guihong Xu,[a] Tian Ke,*[a] Rongrong Fan,[a] Kaiyuan Tan,[a] Wenjun Zhang,[b] Baogen Su,[a] Zhiguo Zhang,[a, b] Zongbi Bao,[a, b] Qilong Ren,[a, b] and Qiwei Yang*[a, b]

[a] G. H. Xu, T. Ke, R. R. Fang, K. Y. Tan, Prof. B. G. Su, Prof. Z. G. Zhang, Prof. Z. B. Bao, Prof. Q. L. Ren, Prof. Q. W. Yang
Key Laboratory of Biomass Chemical Engineering of Ministry of Education, College of Chemical and Biological Engineering Zhejiang University Hangzhou 310027 (China)
E-mail: yangqw@zju.edu.cn, ketian@zju.edu.cn

[b] Prof. W. J. Zhang, Prof. Z. G. Zhang, Prof. Z. B. Bao, Prof. Q. L. Ren, Prof. Q. W. Yang
Institute of Zhejiang University-Quzhou
Quzhou 324000 (China)

## Experimental Section

### Materials

All starting materials and solvents were commercially available and used without further purification.

### Preparation of Ni(BPZ) and Ni(BDP):

Ni(BPZ) was synthesized as the reported reference with minor modifications[1,2]. Typically, 4,4'-Bi-1H-pyrazole ($H_2BPZ$, 134 mg, 1 mmol) was added in 20 mL of MeCN, then add 2 mL of triethylamine. The mixture was stirred at 333 K for 10 min, $Ni(CH_3COO)_2·2H_2O$ (249 mg, 1 mmol) was added. The solution was stirred at 333 K for 12 h under reflux. The orange precipitate formed was filtered off, washed with MeCN. The obtained sample was exchanged with methanol for 3 days, during which the MeOH was refreshed once a day. Finally, the sample was degassed at 423 K for 12 hours before use.
Ni(BDP) were harvested by a procedure similar with that of Ni(BPZ), only replacing $H_2BPZ$ with 1,4-Di(1H-pyrazol-4-yl)benzene ($H_2BDP$, 210 mg, 1 mmol).

### Preparation of Zn(BPZ), Zn(BPZ-2Me) and Zn(BDP):

Zn(BPZ) was synthesized as the reported reference with minor modifications[1]. Typically, $H_2BPZ$ (134 mg, 1 mmol) and sodium methoxide (108 mg, 2 mmol) was added in 100 mL of MeOH. After stirring at 318 K for 30 min, $Zn(CH_3COO)_2·2H_2O$ (220 mg, 1 mmol) was added. The mixture was left under stirring at room temperature for 12 h. A white precipitate was obtained, filtered off, washed twice with MeOH, and dried in air. The sample was degassed at 423 K for 12 hours before use.
Zn(BPZ-2Me) and Zn(BDP) were harvested by a procedure similar with that of Zn(BPZ), only replacing $H_2BPZ$ with 3,3'-Dimethyl-4,4'-Bi-1H-pyrazole ($H_2BPZ$-2Me, 162 mg, 1 mmol) or $H_2BDP$ ( 210 mg, 1 mmol).

### Preparation of Co(BPZ):

Co(BPZ) was synthesized as the reported reference with minor modifications[1]. Typically, $H_2BPZ$ (134 mg, 1 mmol) was dissolved in 30 mL of DMF. Then, $Co(CH_3COO)_2·4H_2O$ (249 mg, 1 mmol) was added. The mixture was left under stirring in a high-pressure glass tube at 120 °C for 24 h, a violet precipitate was obtained, filtered off, washed with hot dichloromethane, and dried in air. The sample was degassed at 423 K for 12 hours before use.

### Preparation of Cu(BPZ):

Cu(BPZ) was synthesized as the reported reference with minor modifications[1]. Typically, $H_2BPZ$ (134 mg, 1 mmol) was dissolved in 150 mL of MeCN. After heated at 318 K for 15 min with concomitant stirring, $Cu(CH_3COO)_2$ (182 mg, 1 mmol) was added. Then, the mixture was stirred at room temperature for 24 h, a brownish precipitate was obtained, filtered off, washed twice with MeCN, and dried in air. The sample was degassed at 423 K for 12 hours before use.

### Preparation of $Fe_2(BDP)_3$:

$Fe_2(BDP)_3$ was synthesized as the reported reference[3]. A 100 ml Schlenk flask was charged with 1059 mg (3 mmol) of Iron acetylacetonate ($Fe(acac)_3$), 210 mg (1 mmol) of $H_2BDP$ and a magnetic stir bar. 15 mL of dry, degassed *N*, *N*-dimethylformamide (DMF) was added to the Schlenk flask via cannula transfer. The reaction was refluxed under nitrogen for 18 hours. The black microcrystalline precipitate was collected on a Buchner funnel. This material was heated in dimethyl sulfoxide (DMSO) at 100°C for 8 hours, and the DMSO was decanted. The material was then washed 5 more times with heated DMSO and similarly six more times with DMF heated

to 100°C and six more times with methylene chloride heated to 70°C. The sample was degassed at 453 K for 24 hours before use.

**Characterization**

Powder X-ray diffraction (PXRD) data were collected on a Rigaku Miniflex 600 diffractometer (Cu $K_\alpha$, λ = 1.540598 Å) with a scan speed of 10.0°/min. The range of 2*θ* was from 3° to 40°. The thermal gravimetric analysis (TGA) was performed on Mettler TGA/DSC 3+. Experiments were going on an alumina pan under nitrogen flow with a heating rate of 10 K/min from 303 K to 1073 K. The MAS solid-state NMR spectra were collected on a Bruker Avance NEO 600WB ($^1$H at 600.41MHz, $^{19}$F at 564.89 MHz) at ambient temperature. The field emission scanning electron microscopy (FESEM) images were taken on a HITACHI SU8010 instrument with a cold field emission gun operating.

**Gas adsorption measurements**

Sorption isotherms of tetrafluoromethane ($CF_4$, 99.999%) and hexafluoroethane ($C_2F_6$, 99.999%) at 273 K, 298 K and 313K, and nitrogen ($N_2$, 99.999%) at 77 K were measured by Micromeritics 3flex surface area analyzer. Around 80 mg activated sample is used for each experiment. Kinetic sorption measurements were conducted by Hiden IGA001 at 3 kPa of $C_2F_6$ and 97 kPa of $CF_4$. Around 60 mg activated sample is used for each experiment. Water ($H_2O$) adsorption experiments were conducted by BELSORP MAX X at 298 K.

**Fitting of pure component isotherms**

The adsorption isotherms of $C_2F_6$ in employed materials were fitted using dual-site Langmuir-Freundlich (DSLF) model.

$$q = q_{A,\ sat}\frac{b_A p^{v_A}}{1 + b_A v_A} + q_{B,\ sat}\frac{b_B p^{v_B}}{1 + b_B v_B} \tag{1}$$

The adsorption isotherms of $CF_4$ in employed materials were fitted using single-site Langmuir-Freundlich (LF) model.

$$q = q_{sat}\frac{b p^{v}}{1 + b v} \tag{2}$$

Here, $p$ is the pressure of the bulk gas at equilibrium with the adsorbed phase (kPa), $q$ is the adsorbed amount per mass of adsorbent (mmol/g), $q_{A,sat}$ and $q_{B,sat}$ are the saturation capacities of site A and B (mmol/g), $b_A$ and $b_B$ are the affinity coefficients of site A and B (kPa$^{-1}$), and $v_A$ and $v_B$ represent the deviations from an ideal homogeneous surface. The parameters that were obtained from the fitting isotherms are provided in Table S2-S3, respectively.

**Isosteric heat of adsorption**

Firstly, the isotherms of $C_2F_6$ and $CF_4$ in Ni(BPZ) and Zn(BPZ) at 273 K, 298 K and 313 K were fitted using Virial type thermal expression:

$$\ln p = \ln q + \frac{1}{T}\sum_{i=0}^{m} a_i q^i + \sum_{i=0}^{n} b_i q^i \tag{3}$$

Then the experimental isosteric heat of adsorption ($Q_{st}$) values for $C_2F_6$ and $CF_4$ in Ni(BPZ) and Zn(BPZ) were calculated:[4]

$$Q_{st} = -R\left(\frac{\partial \ln p}{\partial\,(1/T)}\right)_{n_a} = R\sum_{i=0}^{m} a_i q^i \tag{4}$$

Where $p$ is the pressure described in kPa, $q$ is the adsorption capacity in mmol $g^{-1}$, $T$ is the temperature in K, $a_i$ and $b_i$ are Virial coefficients, $m$ and $n$ are the numbers of coefficients used to describe the isotherms. $Q_{st}$ is the coverage dependent enthalpy of adsorption, and $R$ is the universal gas constant. The related parameters were derived from the fitting of experimental gas adsorption isotherms at different temperatures (as shown in Figure. S17-20). The fitting was carried out by using the Nonlinear Curve Fit with the Multi-Data Fit Mode of global fit in the OriginPro software, Fitting code. The parameters are provided in Table S4.

**IAST calculation of adsorption selectivity**

The Ideal Adsorbed Solution Theory (IAST)[5] selectivity for $C_2F_6/CF_4$ separation is defined by

$$S_{ads} = \frac{q_1/q_2}{p_1/p_2} \quad (5)$$

Where $q_1$ and $q_2$ are the molar loadings in the adsorbed phase in equilibrium with the bulk gas phase with partial pressures $p_1$ and $p_2$, which are calculated by fitting the experimental gas adsorption isotherms using the dual-site Langmuir-Freundlich model or single-site Langmuir-Freundlich model (as shown in the Fitting of pure component isotherms)

**Computational Details**

The surface electrostatic potential of Ni(BPZ) and Zn(BPZ) were calculated using the Energy task in DMol3 module[6,7] of Materials Studio, the electron density and electrostatics properties of the framework were calculated, with the density field being the total density and the potential field being the electrostatic potential field. The molecular surface electrostatic potential was also calculated using the DMol3 code of Materials Studio for $C_2F_6$ and $CF_4$, respectively.

The GCMC simulations were performed using the Locate task in Sorption module of Materials Studio. A combination of the Lennard-Jones 6−12 (L-J) potentials and the electrostatic potentials were used to compute the intermolecular interactions. The L-J parameters were taken from the universal force field (UFF), and the partial atomic charges were obtained with charge equilibration method ($Q_{eq}$) methods. The $CF_4$ and $C_2F_6$ gas molecules models were optimized by the DMol3 module of Materials Studio. The structures of MOFs were treated as rigid with atoms frozen at their crystallographic positions during the process of GCMC simulations. A cutoff radius of 15.5 Å was applied to calculate the nonbonded interactions, and the electrostatic interactions were handled using the Ewald summation method. All GCMC simulations included a $1.0 \times 10^6$ production steps and a $1.0 \times 10^5$ maximum loading steps.

The static binding energy was calculated using the combination of first-principle density functional theory (DFT) and plane-wave ultrasoft pseudopotential implemented in the Materials Studio, CASTEP module.[8] A semi-empirical addition of dispersive forces to conventional DFT was included in the calculation to account for van der Waals interactions. Calculations were performed under the generalized gradient approximation (GGA) with Perdew-Burke-Ernzerhof (PBE) exchange correlation. A cutoff energy of 544 eV while a 1×1×2 k-point mesh for Ni(BPZ) and 2×2×1 k-point mesh for Zn(BPZ) were found to be enough for the total energy to converge within $1\times10^{-6}$ eV $atom^{-1}$, the calculation error are within 0.001 Å. The initial adsorption structure of gas molecules in MOFs was determined by GCMC simulations, and optimized by DFT calculations. Then the energy of adsorption structure ($E_{host+guest}$), the guest molecule ($E_{guest}$ or $E_{single\ guest}$) and the host framework ($E_{host}$) were calculated using the Energy task in CASTEP module, respectively. The static binding energy (at T = 0 K) was then calculated:

$$\Delta E_{host-guest} = E_{host} + E_{guest} - E_{host+guest} \quad (6)$$

$$\Delta E_{guest-guest} = E_{guest} - N \times E_{single\ guest} \quad (7)$$

**Breakthrough tests**

The breakthrough experiments were carried out in a dynamic gas breakthrough equipment. All experiments were conducted using a stainlesss teel column (4.6 mm inner diameter × 50 mm) and the mass packed in the sample holder was: Ni(BPZ) (196 mg) and Zn(BPZ) (266 mg). Activated MOF powder was packed into stainless steel column under pure $N_2$ atmosphere. The breakthrough experiments were carried out at flow rates of 1 mL/min for $C_2F_6/CF_4$ 3/97 (*v/v*) mixtures and 3 mL/min for $C_2F_6/CF_4$ 1/99 (*v/v*) mixtures. Outlet gas from the column was monitored using gas chromatography (GC-2010, SHIMADZU) with a thermal conductivity detector (TCD). The actual gas uptake capacity obtained by the desorption process was calculated using the following equation:

$$n_i = F_i(t_2 - t_1) - \int_{t_1}^{t_2} FC_i dt \quad (8)$$

where $n_i$ was the uptake capacity of the gas component *i*, $F_i$ was flow rate of gas *i* in the inlet, *F* was the total outlet flow rate which was calculated based on the flow rate of He and the concentration of each gas component, $C_i$ was the concentration of gas *i* in the outlet gas mixture, $t_1$ was the initial gas-detected time, $t_2$ was the final gas-detected time.

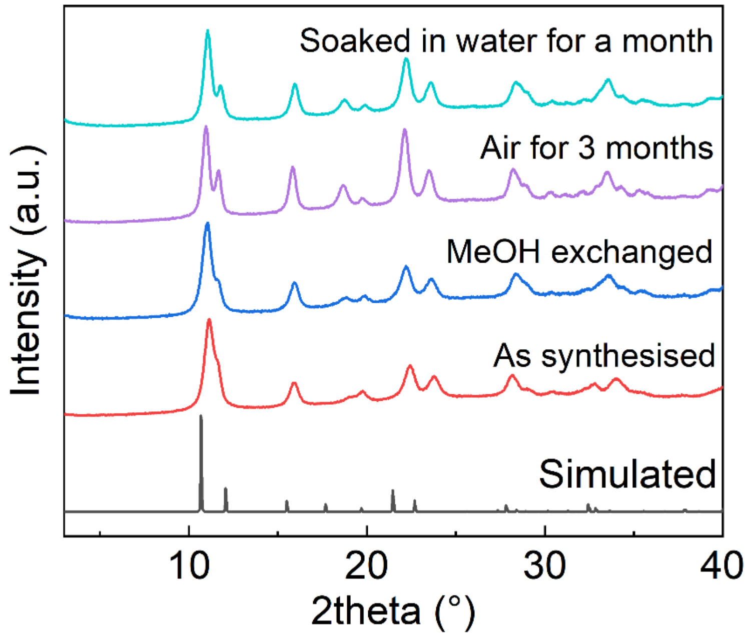


**Figure S1.** Powder X-ray diffraction patterns of Ni(BPZ).

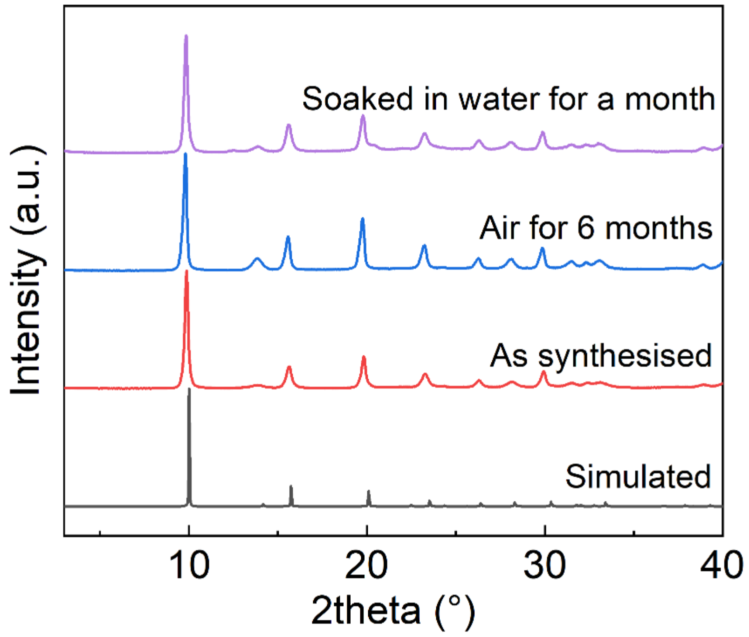


**Figure S2.** Powder X-ray diffraction patterns of Zn(BPZ).

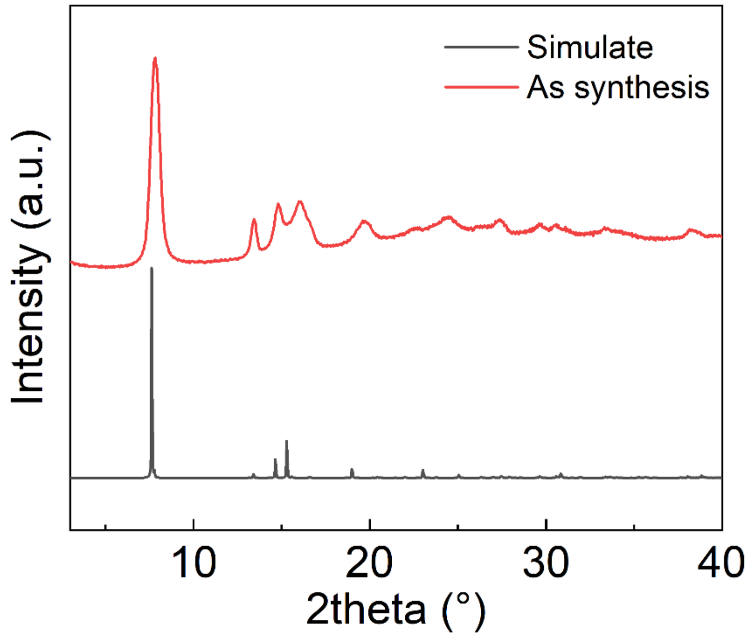


**Figure S3.** Powder X-ray diffraction patterns of Zn(BDP).

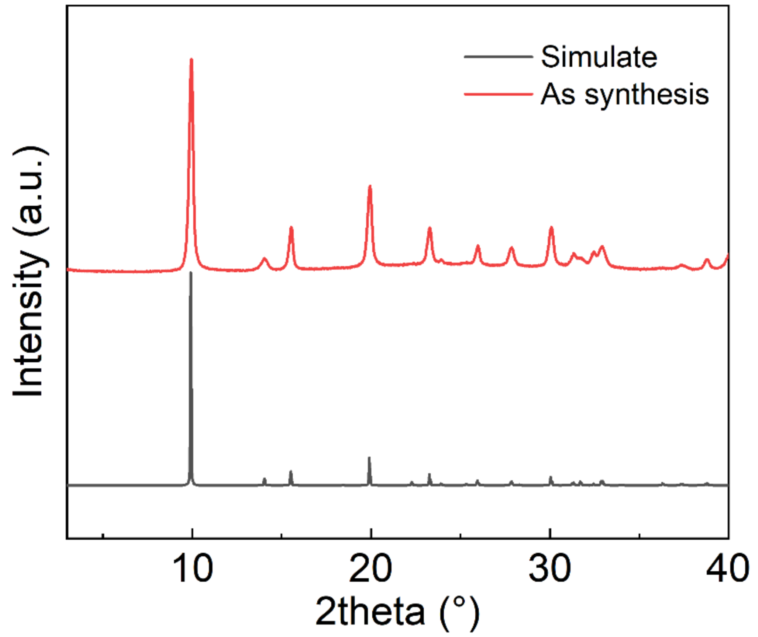


**Figure S4.** Powder X-ray diffraction patterns of Zn(BPZ-2Me).

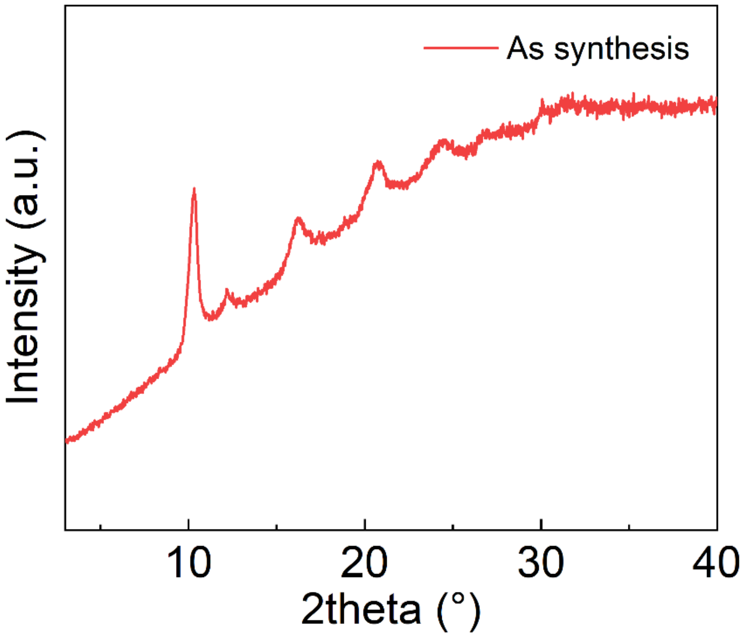


**Figure S5.** Powder X-ray diffraction patterns of Co(BPZ) (contaminated with fluorescence).

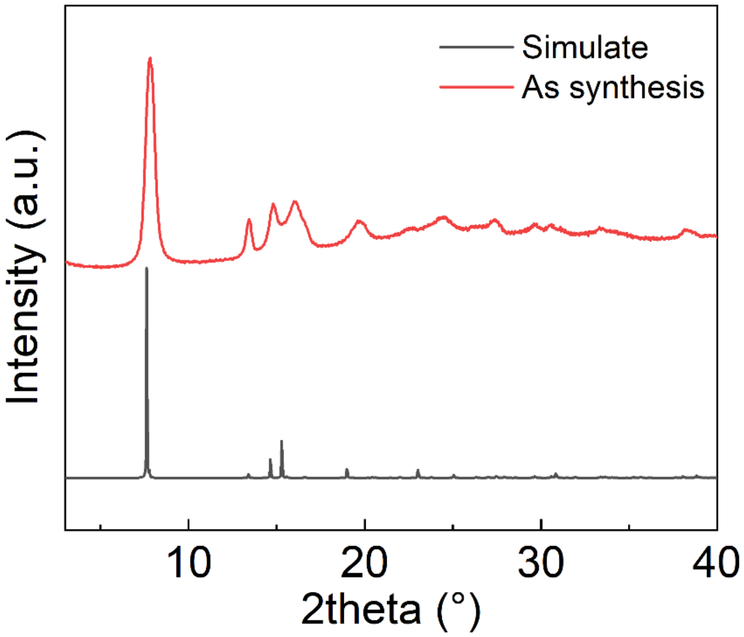


**Figure S6.** Powder X-ray diffraction patterns of Ni(BDP).

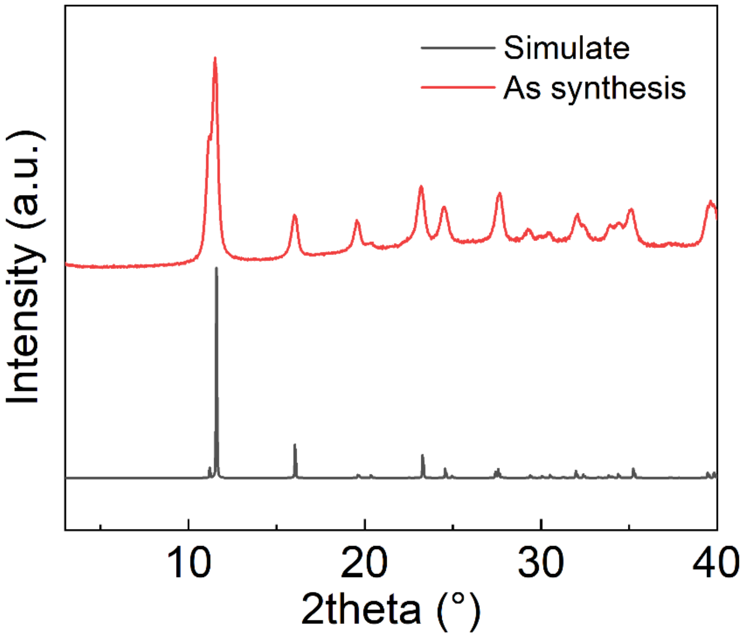


**Figure S7.** Powder X-ray diffraction patterns of Cu(BPZ).

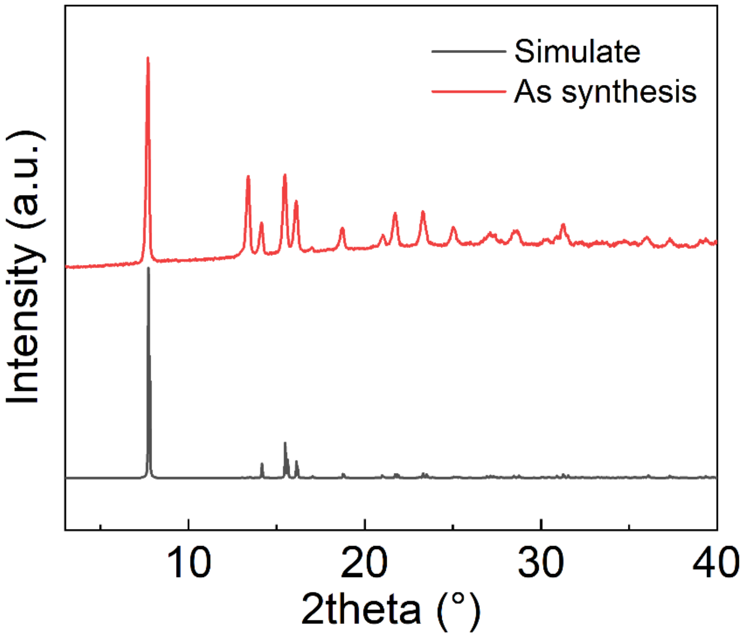


**Figure S8.** Powder X-ray diffraction patterns of $Fe_2(BDP)_3$.

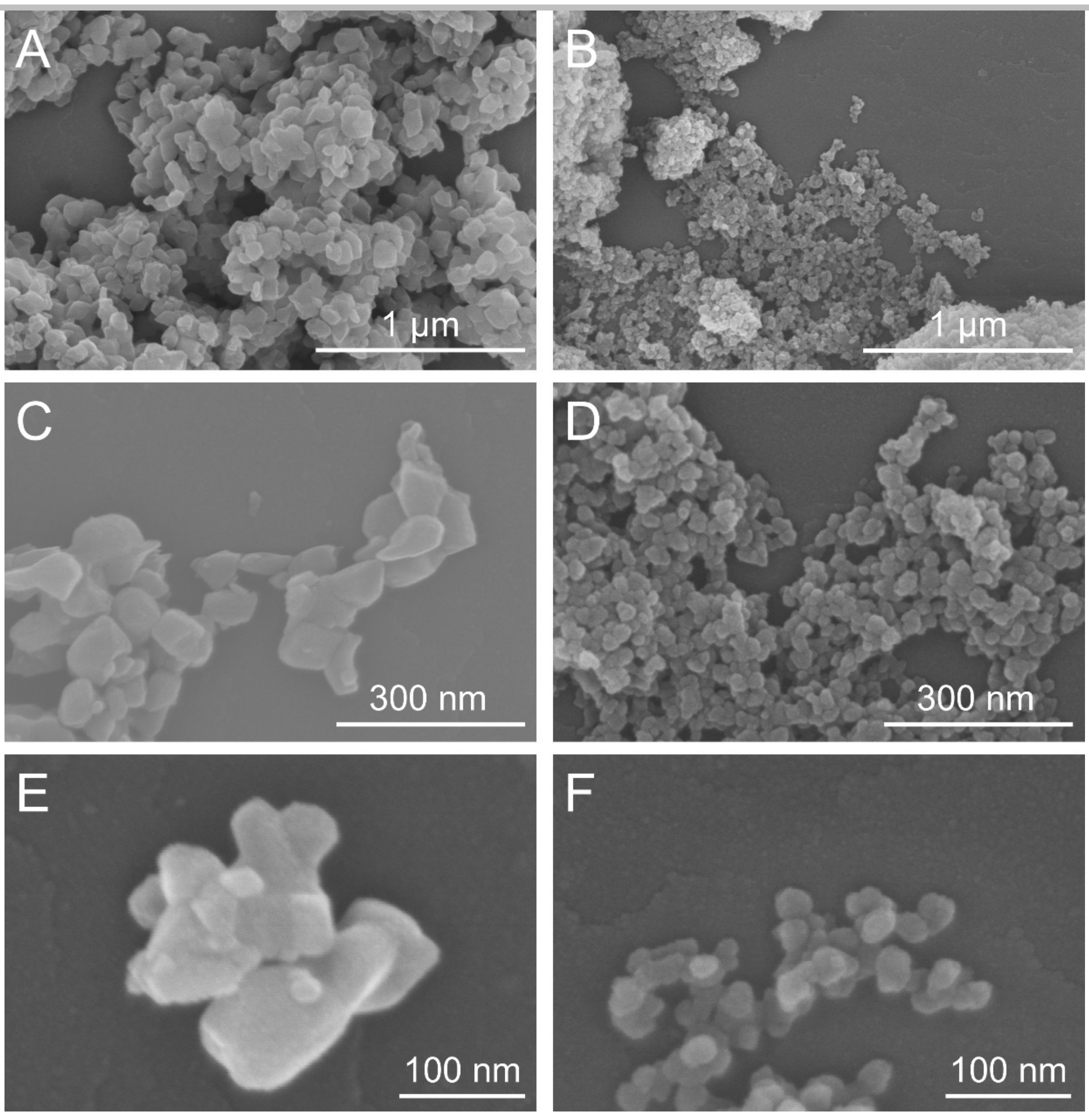


**Figure S9.** The SEM images of Zn(BPZ) (left column: A, C and E) and Ni(BPZ) (right column: B, D and F).

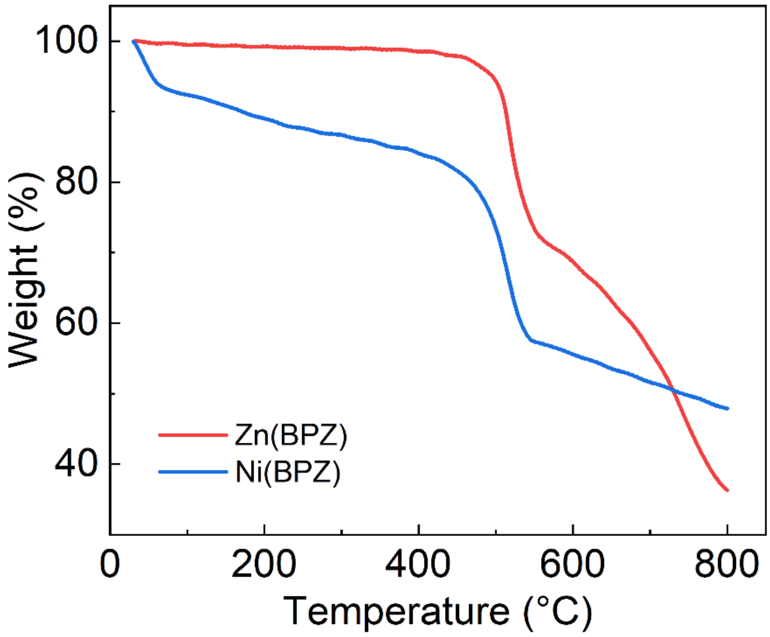


**Figure S10.** TGA curves of the Ni(BPZ) and Zn(BPZ) powder samples, respectively.

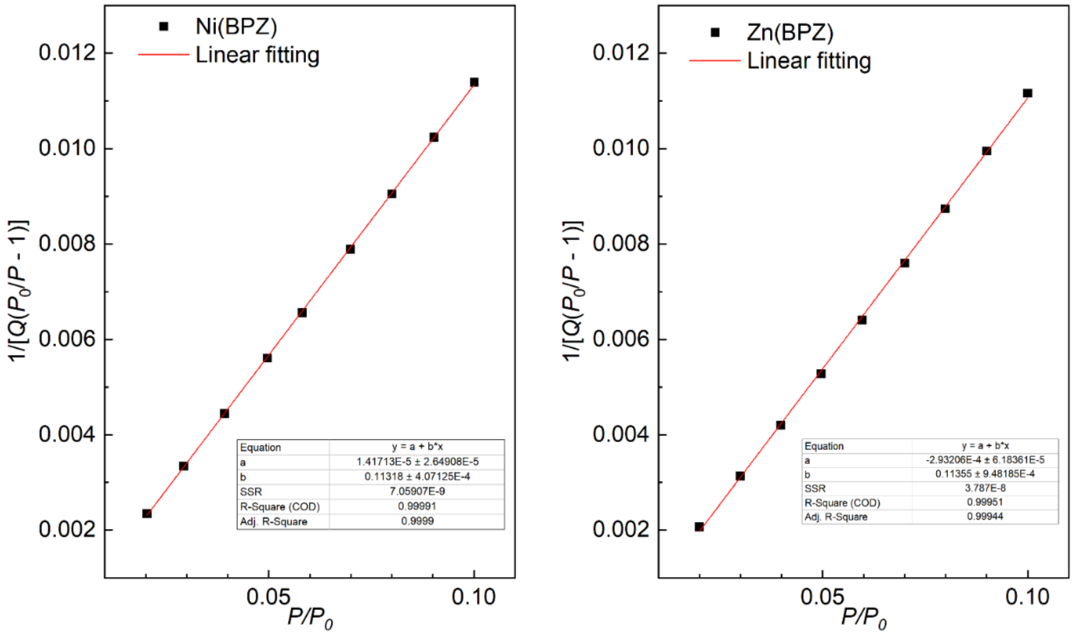


**Figure S11.** Calculated BET surface area of Ni(BPZ) (left) and Zn(BPZ) (right) by $N_2$ adsorption isotherm at 77 K, respectively.

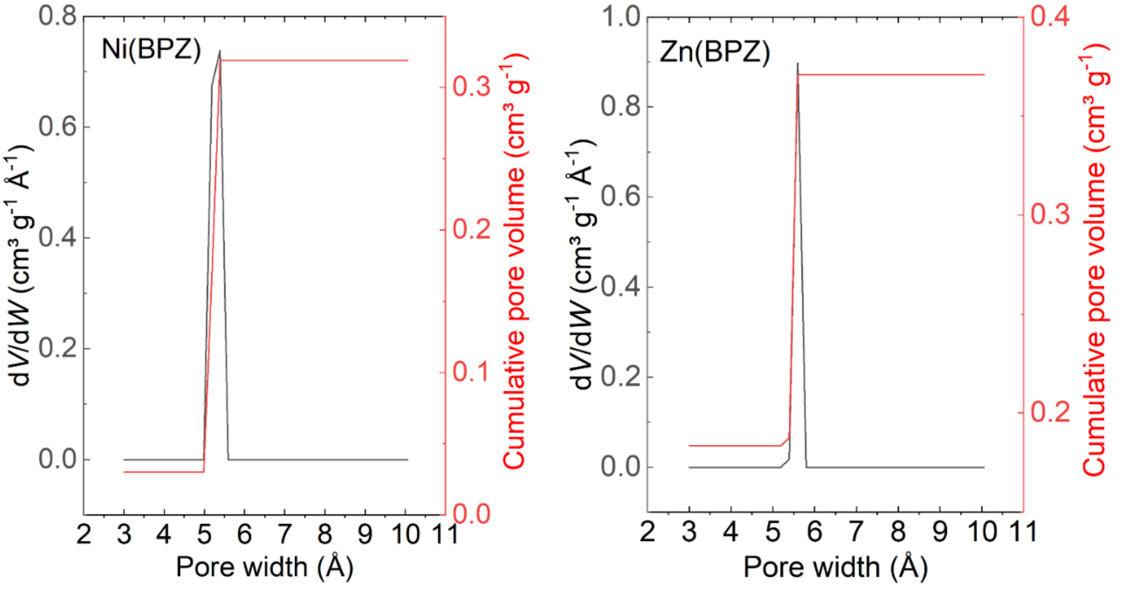


**Figure S12.** The pore size distribution of Ni(BPZ) (left) and Zn(BPZ) (right), respectively.

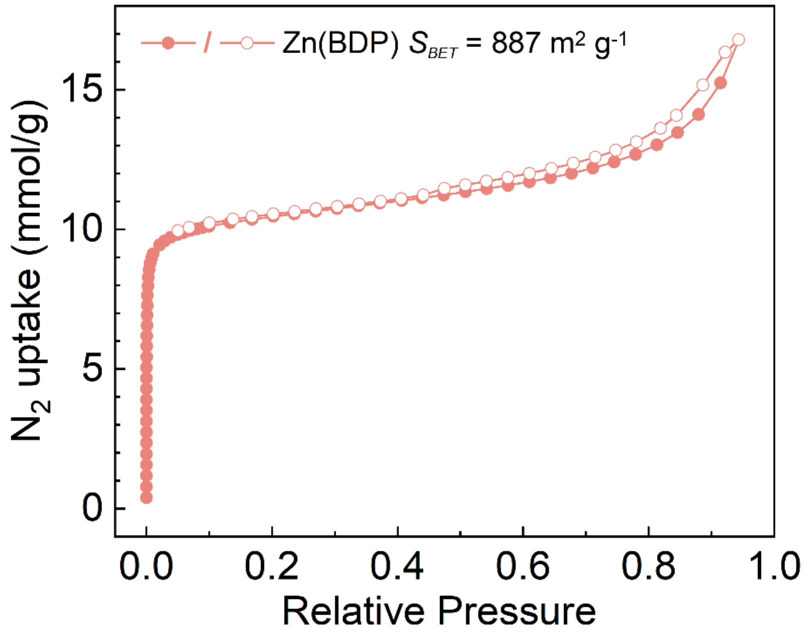


**Figure S13.** The $N_2$ adsorption isotherms of Zn(BDP) at 77 K.

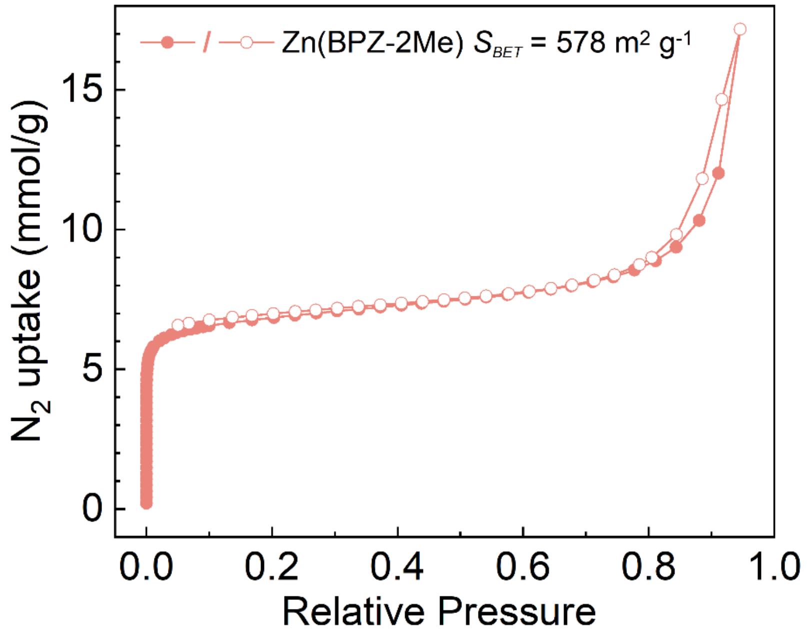


**Figure S14.** The $N_2$ adsorption isotherms of Zn(BPZ-2Me) at 77 K.

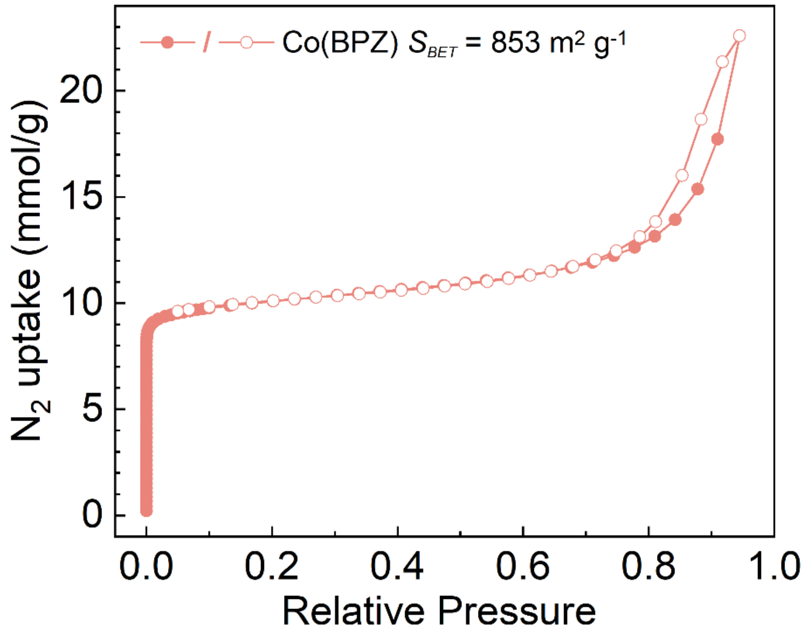


**Figure S15.** The $N_2$ adsorption isotherms of Co(BPZ) at 77 K.

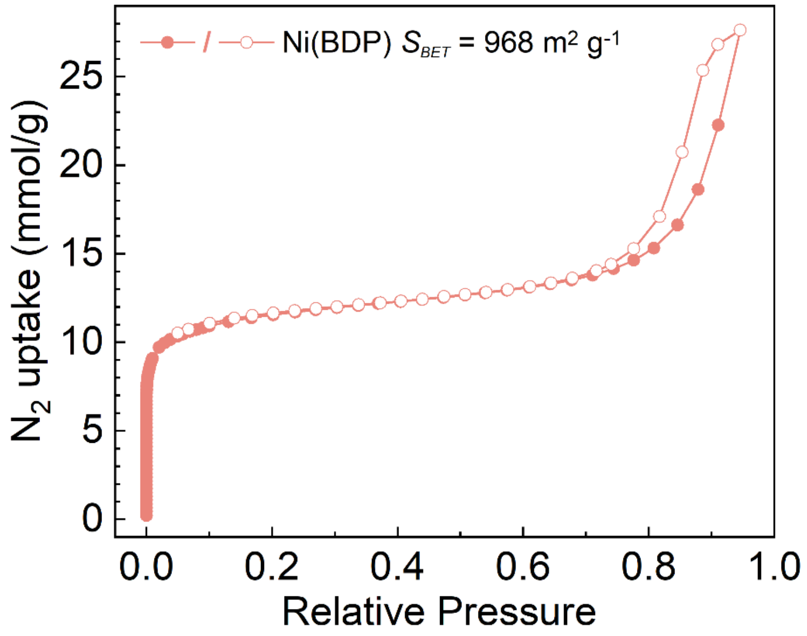


**Figure S16.** The $N_2$ adsorption isotherms of Ni(BDP) at 77 K.

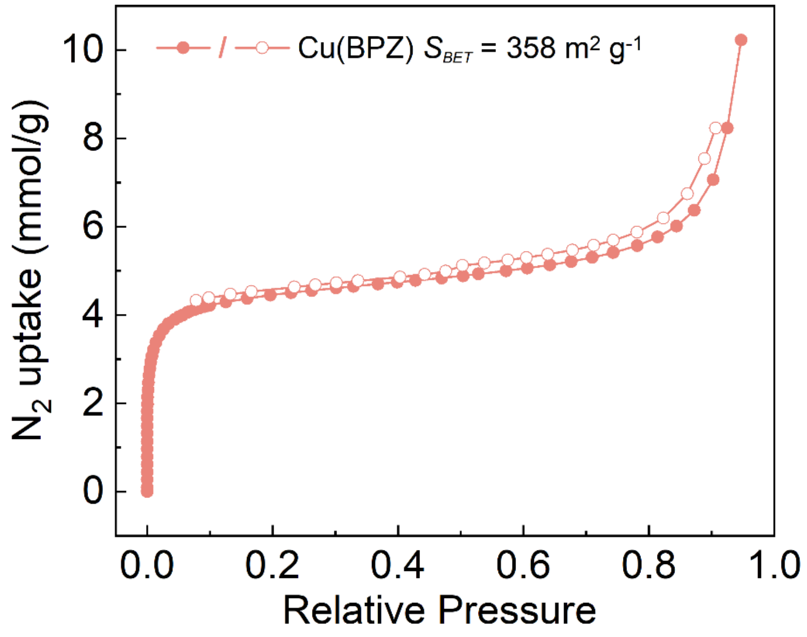


**Figure S17.** The $N_2$ adsorption isotherms of Cu(BPZ) at 77 K.

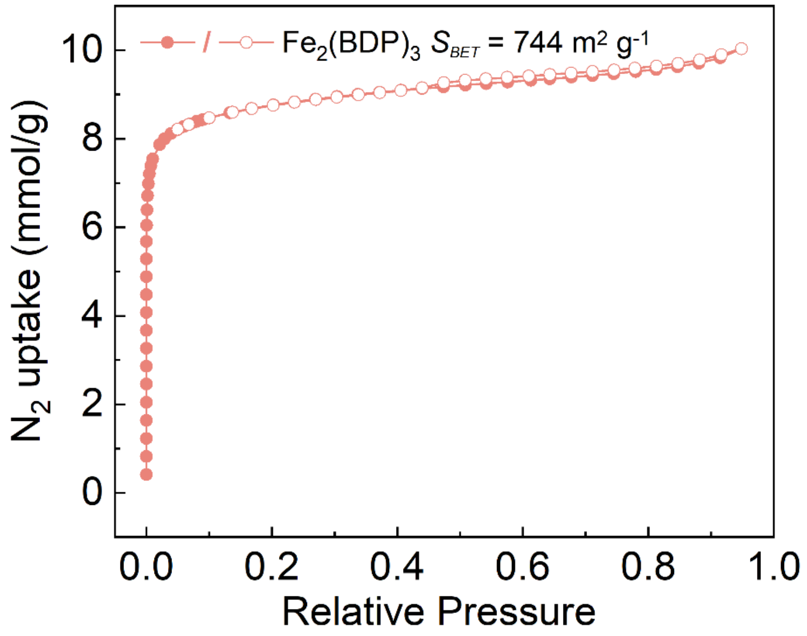


**Figure S18.** The $N_2$ adsorption isotherms of $Fe_2(BDP)_3$ at 77 K.

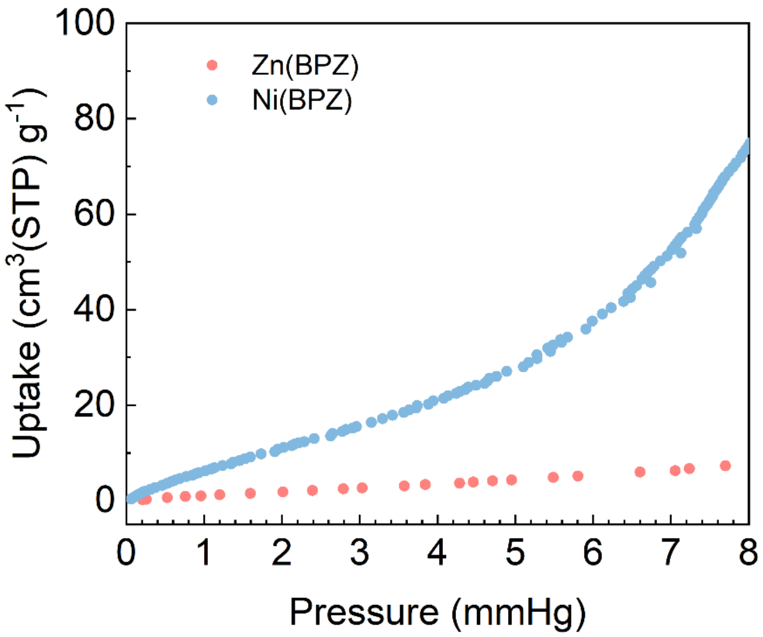


**Figure S19.** Water ($H_2O$) adsorption isotherms of Ni(BPZ) and Zn(BPZ) at 298 K.

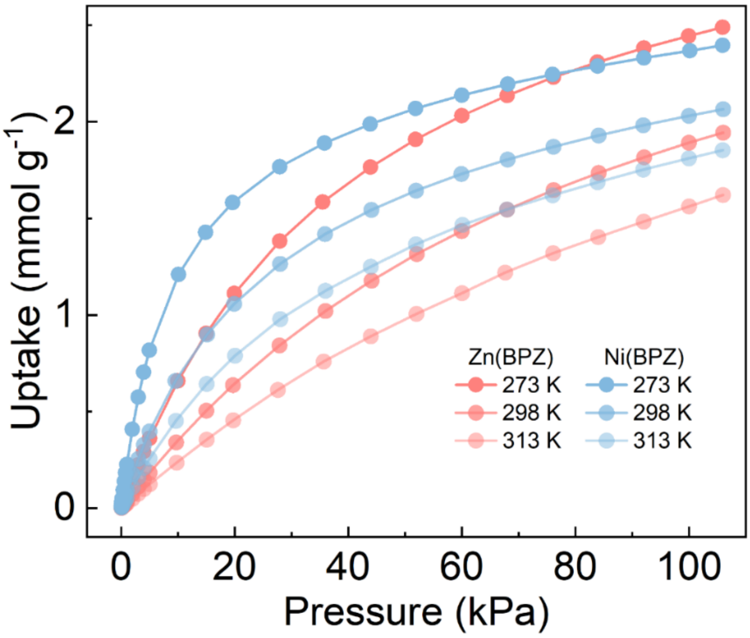


**Figure S20.** The single component isotherms of $CF_4$ on Ni(BPZ) and Zn(BPZ) at 273 K, 298 K and 313 K, respectively.

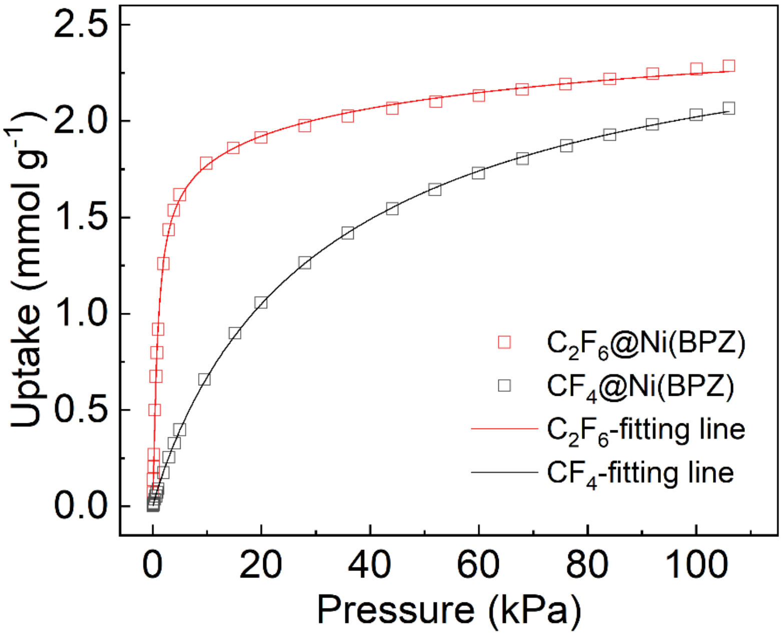


**Figure S21.** $CF_4$ and $C_2F_6$ adsorption data and DSLF fitting line at 298 K on Ni(BPZ), respectively.

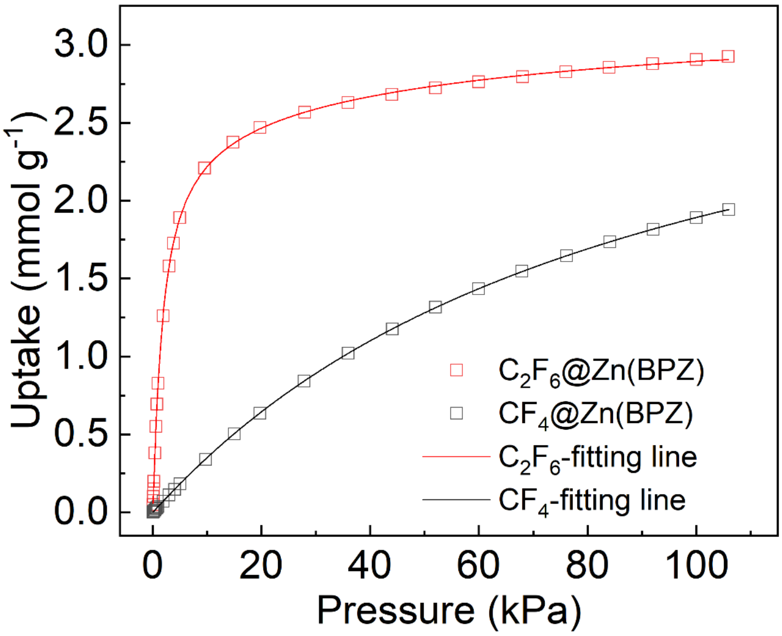


**Figure S22.** $CF_4$ and $C_2F_6$ adsorption data and DSLF fitting line at 298 K on Zn(BPZ), respectively.

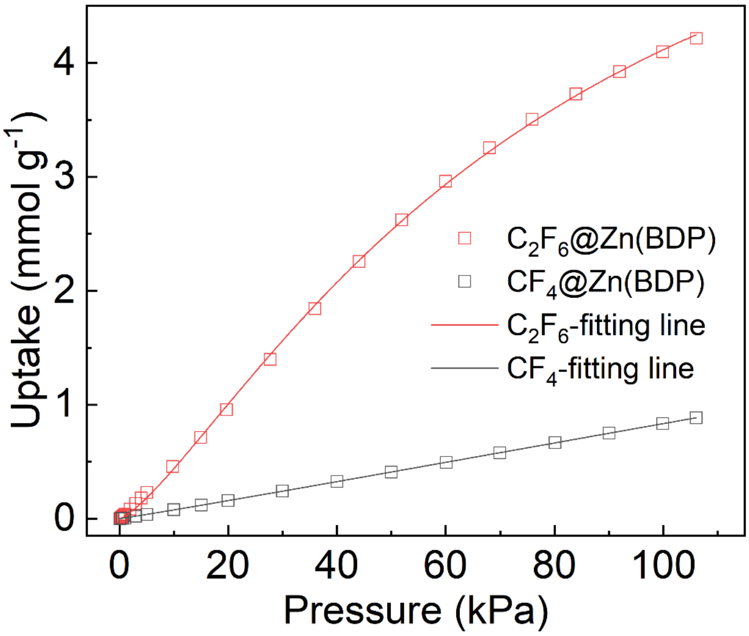


**Figure S23.** $CF_4$ and $C_2F_6$ adsorption data and DSLF fitting line at 298 K on Zn(BDP), respectively.

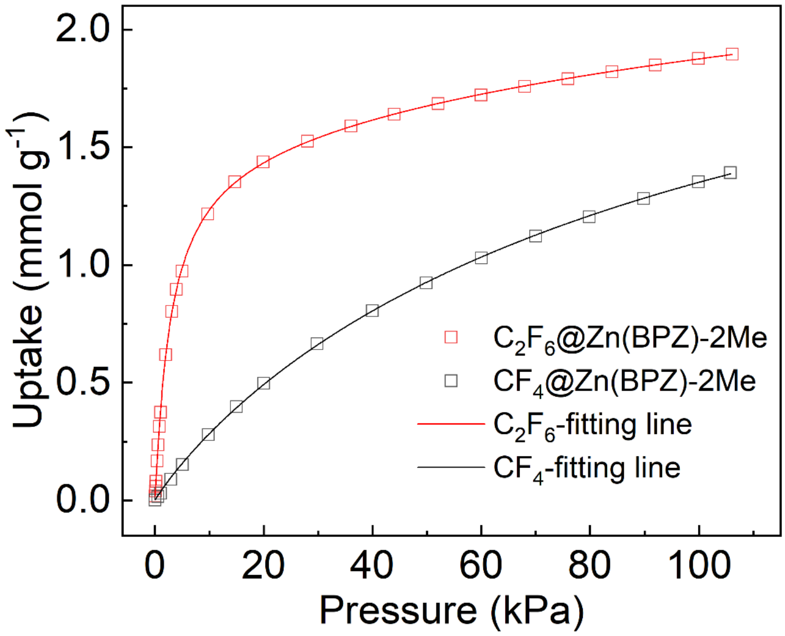


**Figure S24.** $CF_4$ and $C_2F_6$ adsorption data and DSLF fitting line at 298 K on Zn(BPZ)-2Me, respectively.

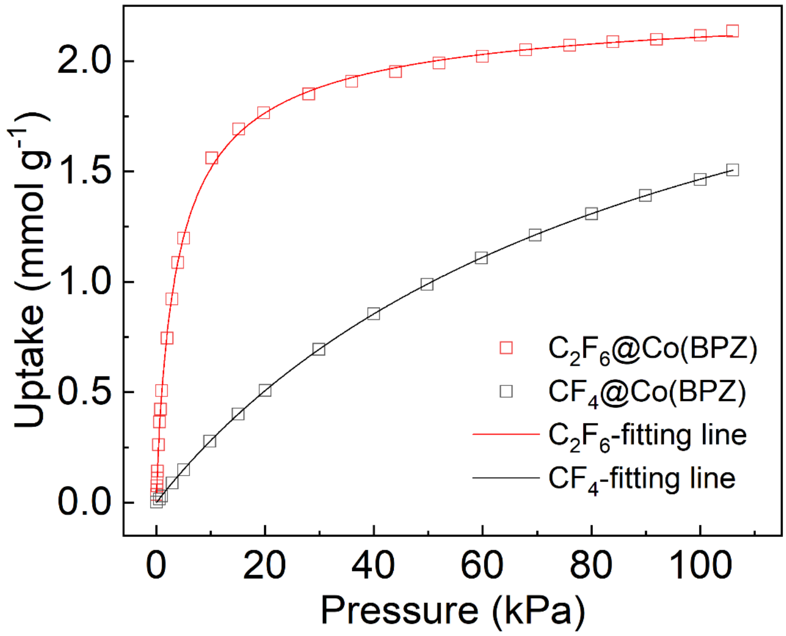


**Figure S25.** $CF_4$ and $C_2F_6$ adsorption data and DSLF fitting line at 298 K on Co(BPZ), respectively.

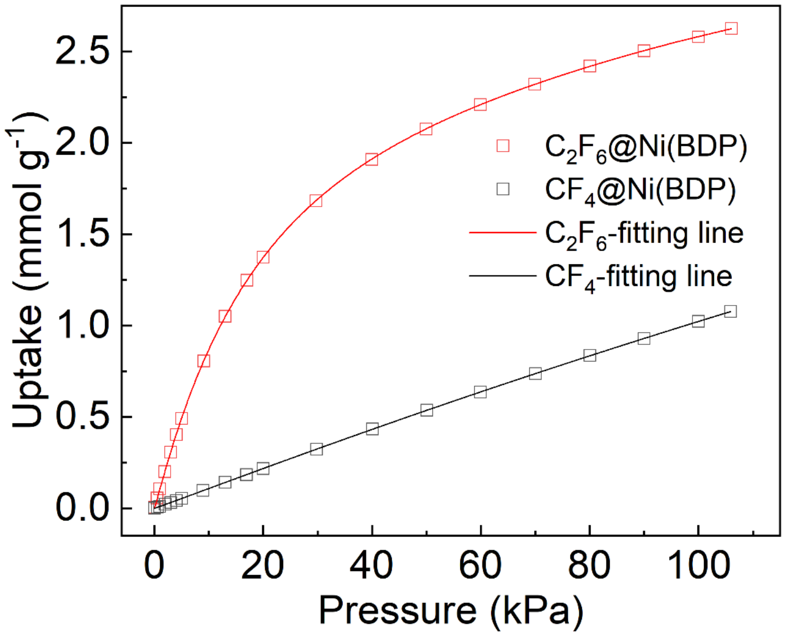


**Figure S26.** $CF_4$ and $C_2F_6$ adsorption data and DSLF fitting line at 298 K on Ni(BDP), respectively.

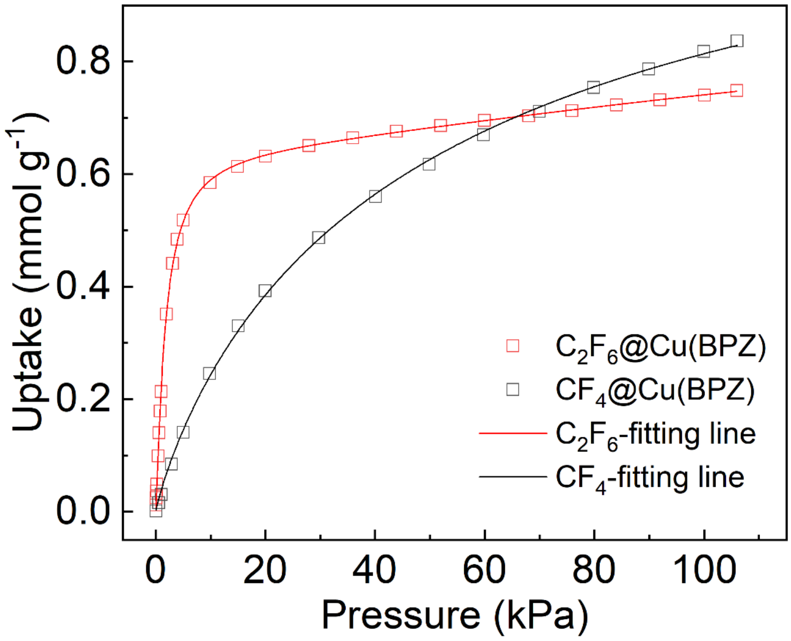


**Figure S27.** $CF_4$ and $C_2F_6$ adsorption data and DSLF fitting line at 298 K on Cu(BPZ), respectively.

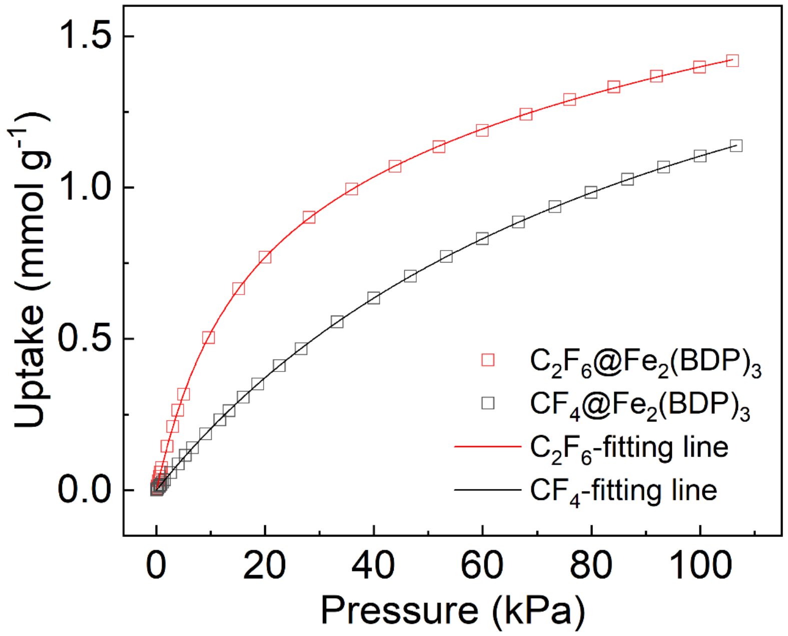


**Figure S28.** $CF_4$ and $C_2F_6$ adsorption data and DSLF fitting line at 298 K on $Fe_2(BDP)_3$, respectively.

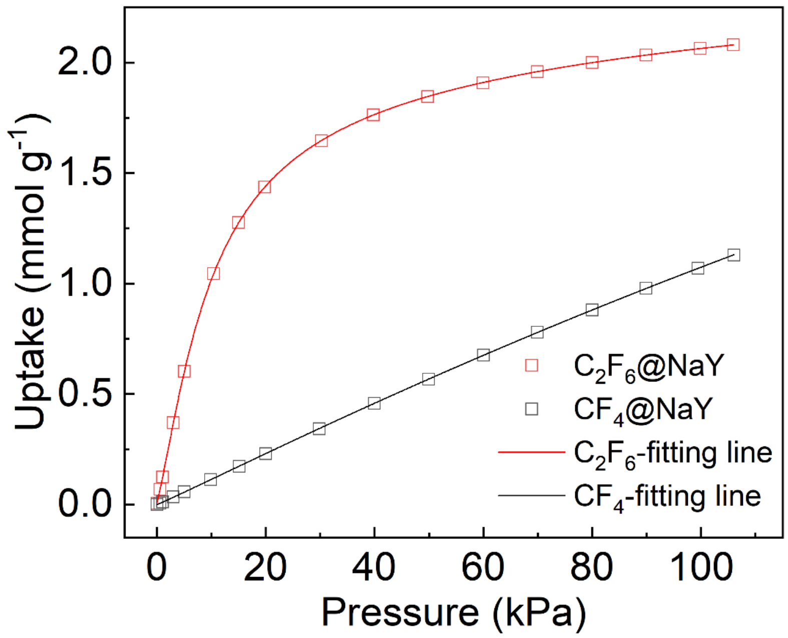


**Figure S29.** $CF_4$ and $C_2F_6$ adsorption data and DSLF fitting line at 298 K on NaY, respectively.

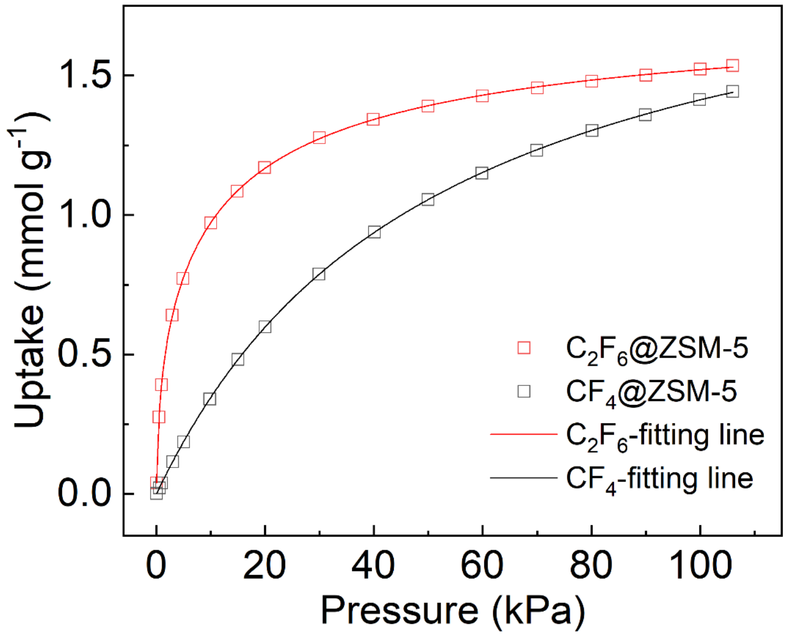


**Figure S30.** $CF_4$ and $C_2F_6$ adsorption data and DSLF fitting line at 298 K on ZSM-5, respectively.

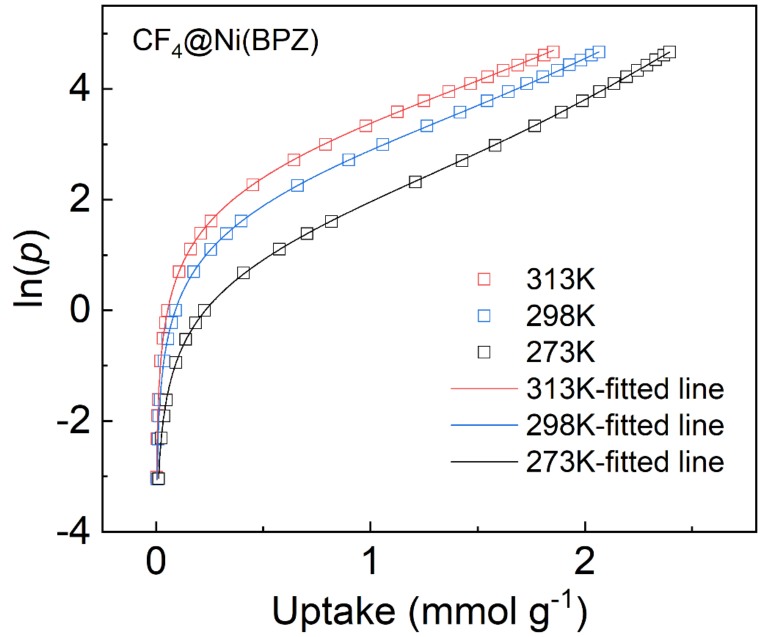


**Figure S31.** The Virial fitting of $CF_4$ adsorption data of Ni(BPZ) at different temperatures.

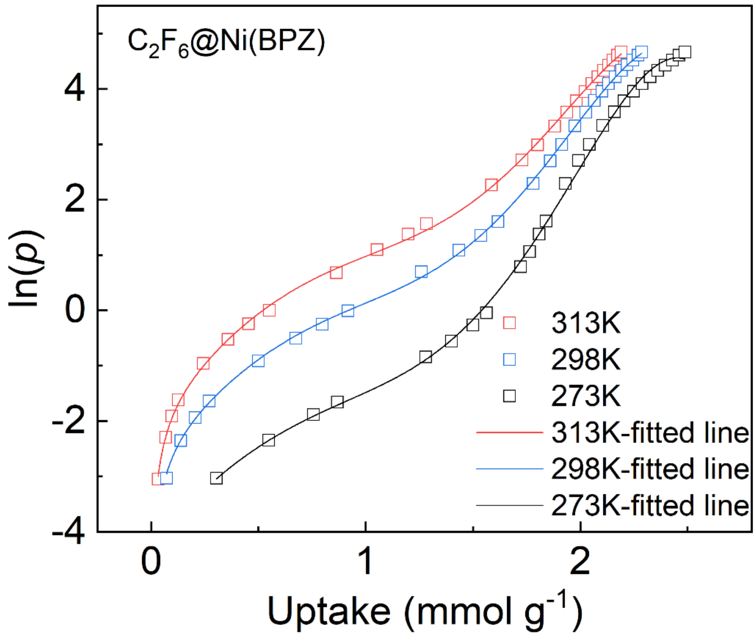


**Figure S32.** The Virial fitting of $C_2F_6$ adsorption data of Ni(BPZ) at different temperatures.

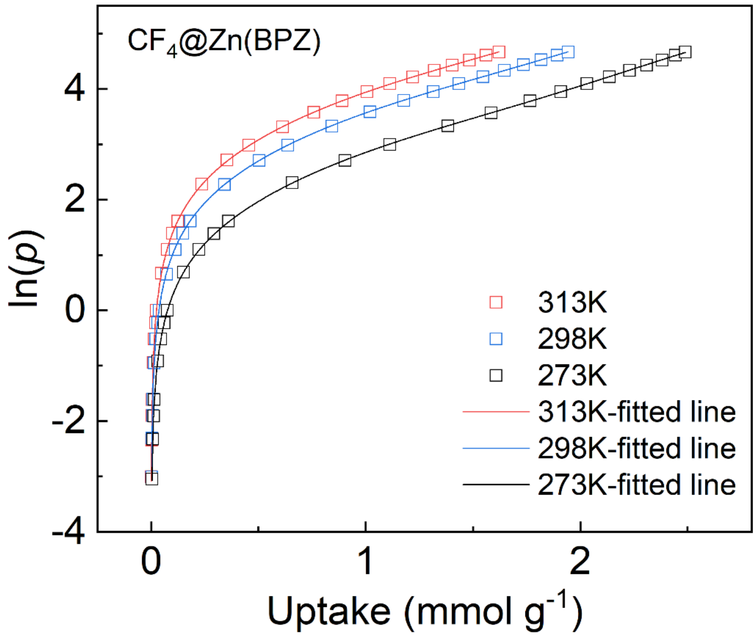


**Figure S33.** The Virial fitting of $CF_4$ adsorption data of Zn(BPZ) at different temperatures.

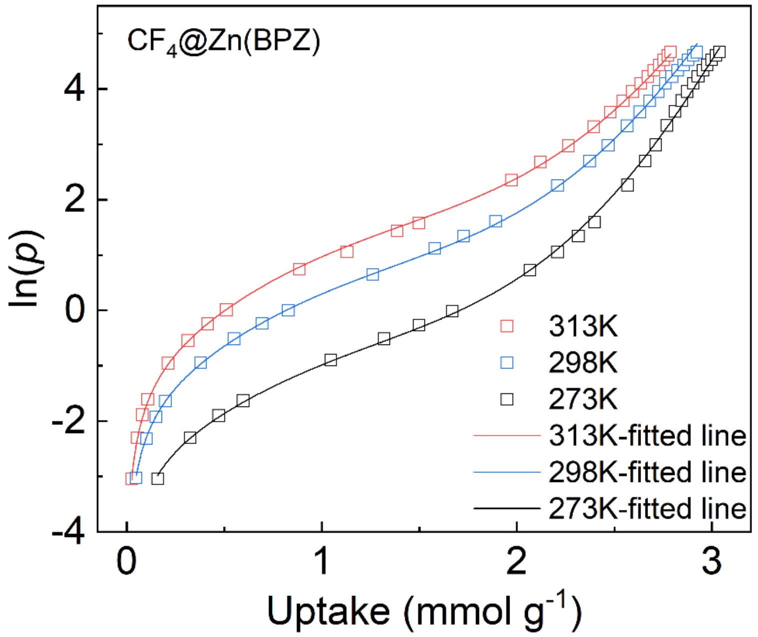


**Figure S34.** The Virial fitting of $C_2F_6$ adsorption data of Zn(BPZ) at different temperatures.

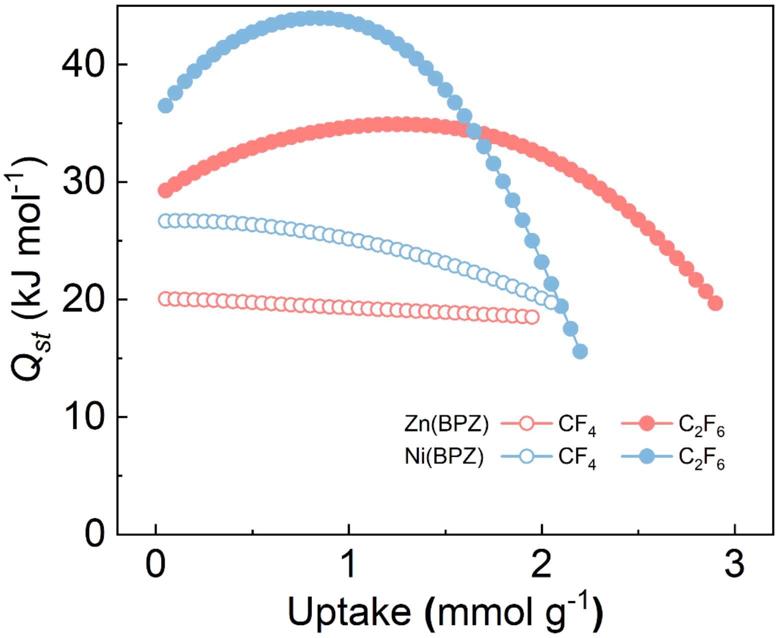


**Figure S35.** Isosteric heat of adsorption ($Q_{st}$) for $C_2F_6$ and $CF_4$ of Ni(BPZ) and Zn(BPZ), respectively.

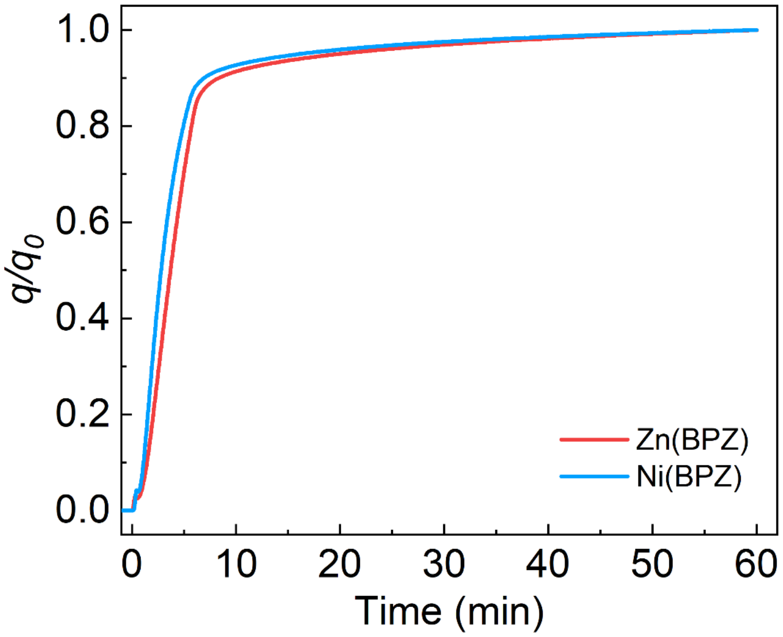


**Figure S36.** Kinetic sorption measurements of $CF_4$ on Ni(BPZ) and Zn(BPZ) at 97 kPa and 298 K, respectively.

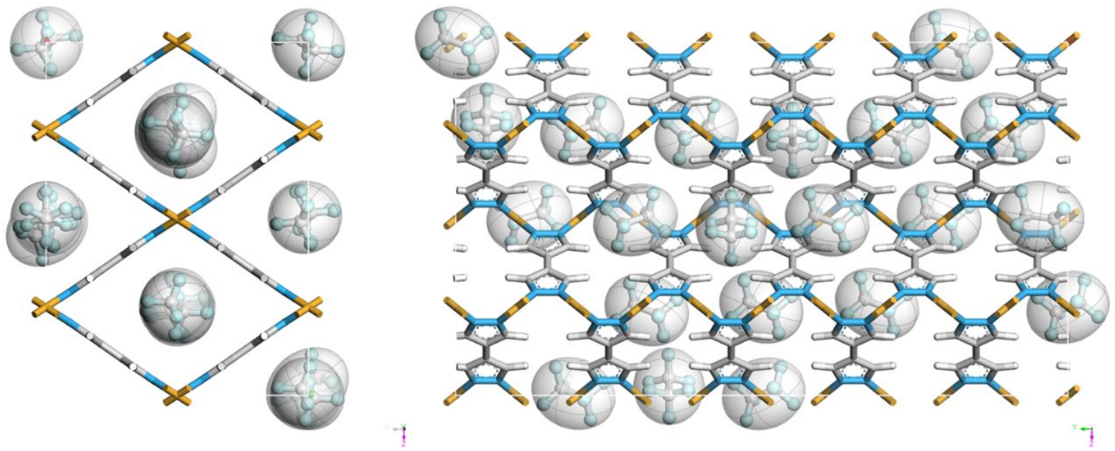

**Figure S37.** The guest inclusion structure of maximum $C_2F_6$ loadings in the supercell of $[Ni(BPZ)]_{40}$ simulated by GCMC.

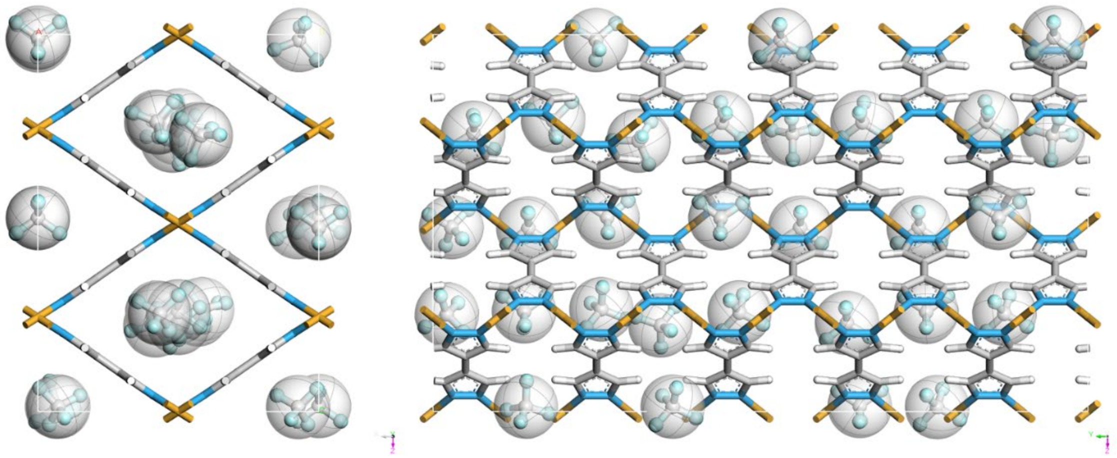

**Figure S38.** The guest inclusion structure of maximum $CF_4$ loadings in the supercell of $[Ni(BPZ)]_{40}$ simulated by GCMC.

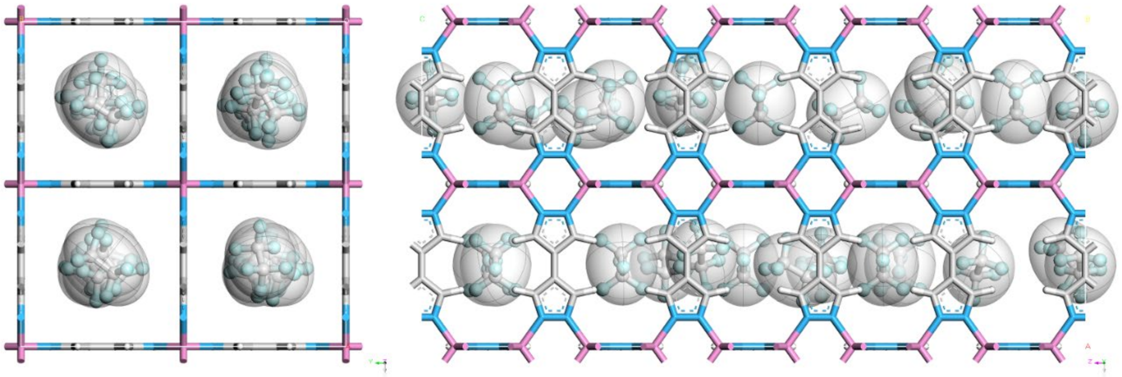

**Figure S39.** The guest inclusion structure of maximum $C_2F_6$ loadings in the supercell of $[Ni(BPZ)]_{40}$ simulated by GCMC.

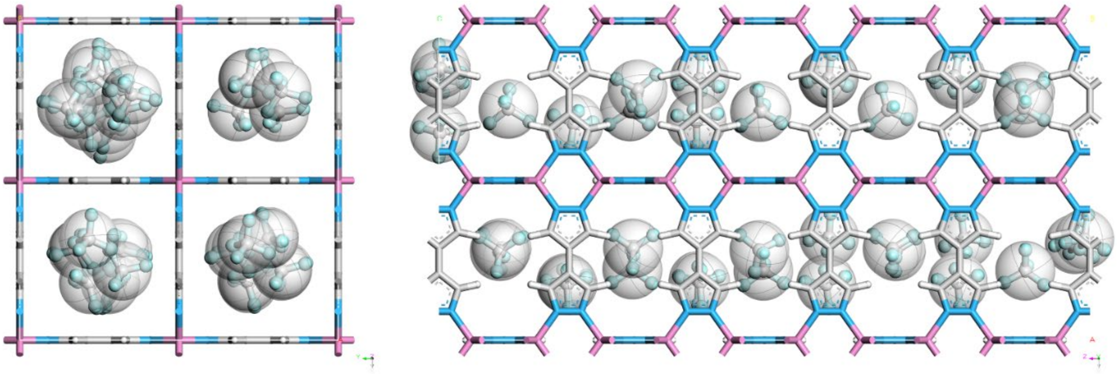

**Figure S40.** The guest inclusion structure of maximum $CF_4$ loadings in the supercell of $[Zn(BPZ)]_{40}$ simulated by GCMC.

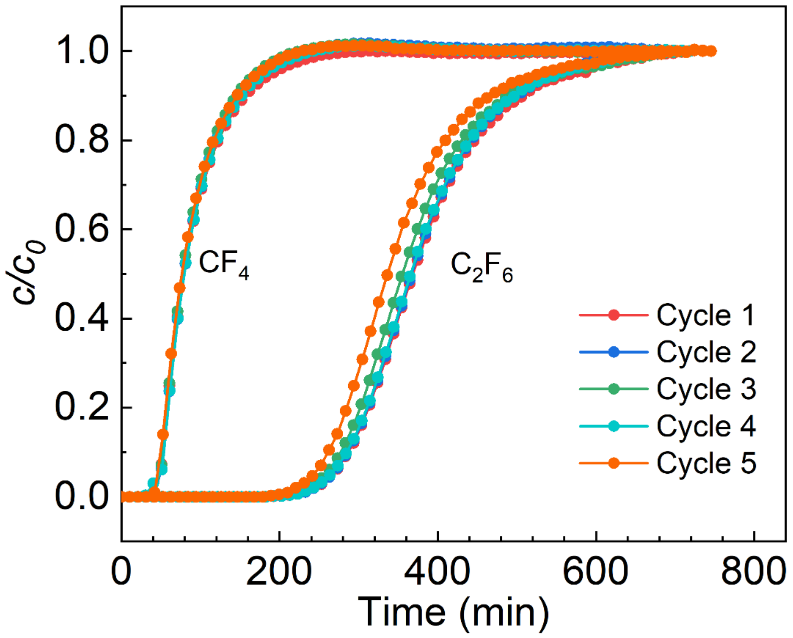


**Figure S41.** Cycling column breakthrough curves of $C_2F_6/CF_4$ 3/97 (v/v) gas mixture on Ni(BPZ) at 298 K and 1 bar with a flow rate of 1 mL $min^{-1}$.

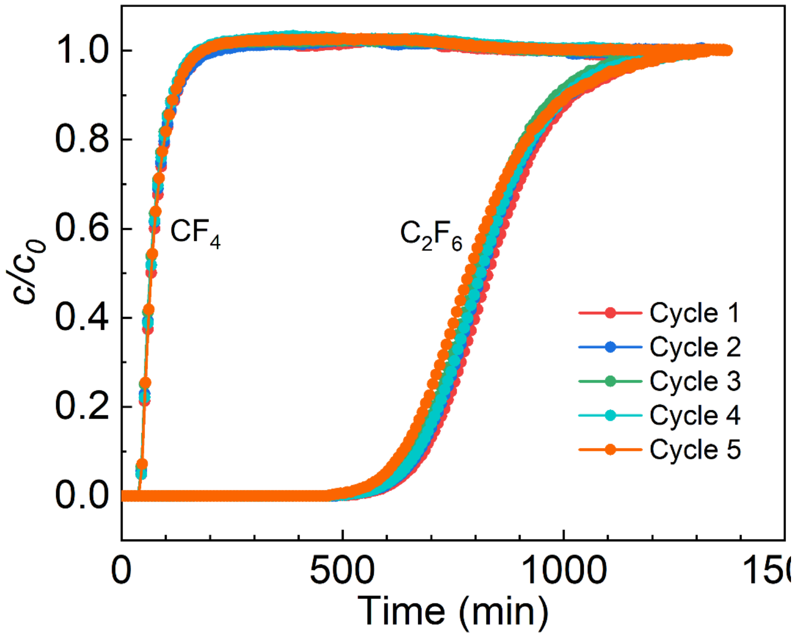


**Figure S42.** Cycling column breakthrough curves of $C_2F_6/CF_4$ 3/97 (v/v) gas mixture on Zn(BPZ) at 298 K and 1 bar with a flow rate of 1 mL $min^{-1}$.

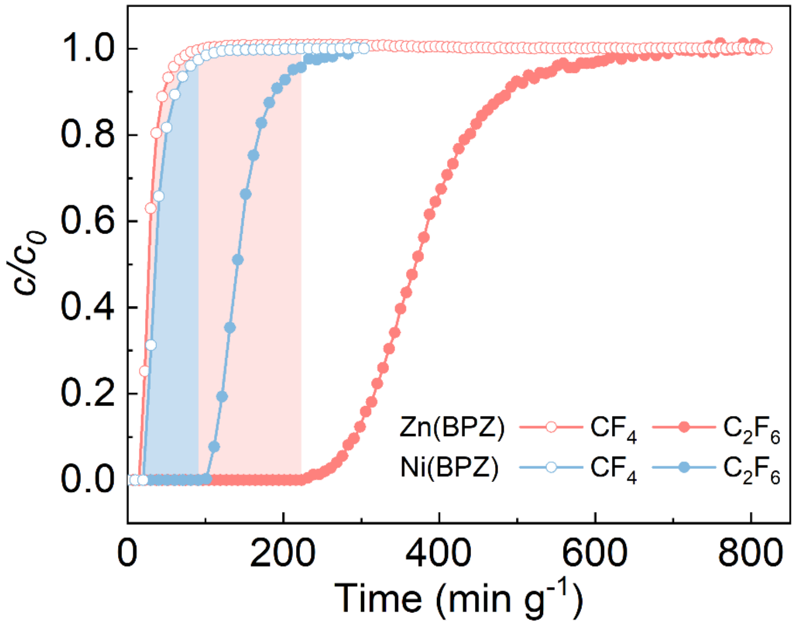


**Figure S43.** Experimental breakthrough curves for $C_2F_6/CF_4$ mixture (1/99, v/v) at 298 K and 1 bar with a flow rate of 3 mL $min^{-1}$ on Ni(BPZ) and Zn(BPZ) columns, respectively.

**Table S1.** The property of PFCs generated from tetrafluoromethane production.[9]

| Property | $CF_4$ | $C_2F_6$ |
|---|---|---|
| Molecular weight/ (g $mol^{-1}$) | 88.01 | 138.01 |
| Boiling point (1 atm)/°C | -128 | -78.2 |
| Critical temperature/°C | -45.6 | 19.7 |
| Critical pressure/MPa | 3.74 | 2.99 |
| Heat of vaporization at boiling point/ (kJ $kg^{-1}$) | 135.9 | 117.0 |
| Kinetic diameter/Å | 4.66 | 5.10 |
| Polarizability×$10^{25}$/$cm^3$ | 38.4 | 68.2 |
| Dipole moment×$10^{18}$/ (esu cm) | 0 | 0 |
| Quadruple moment×$10^{26}$/ (esu $cm^2$) | 0 | 0 |

**Table S2.** The fitting parameters for dual-site Langmuir-Freundlich (DSLF) isotherm model of $C_2F_6$ at 298 K.

| $C_2F_6$ | $q_{A, sat}$ | $b_A$ | $v_A$ | $q_{A, sat}$ | $b_A$ | $v_A$ | $R^2$ |
|---|---|---|---|---|---|---|---|
| Zn(BPZ) | 1.9045 | 0.5209 | 1.2385 | 1.5871 | 0.1263 | 0.5654 | 0.9999 |
| Ni(BPZ) | 1.6543 | 0.1729 | 0.4736 | 1.2472 | 1.2391 | 1.4008 | 0.9996 |
| Co(BPZ) | 1.9030 | 0.3080 | 0.9160 | 0.4352 | 0.1308 | 0.5966 | 0.9996 |
| Cu(BPZ) | 0.6564 | 0.5131 | 1.2139 | 0.2918 | 0.0003 | 1.6108 | 0.9999 |
| Zn(BPZ)-2Me | 1.8737 | 0.0132 | 0.7376 | 1.3710 | 0.3467 | 1.0830 | 0.9999 |
| Zn(BDP) | 5.4858 | 0.0001 | 2.4638 | 1.6554 | 0.0076 | 1.0561 | 0.9999 |
| Ni(BDP) | 2.9573 | 0.0367 | 1.0553 | 0.2055 | 0.0158 | 0.8458 | 0.9999 |
| $Fe_2(BDP)_3$ | 1.8087 | 0.0081 | 0.8466 | 0.9830 | 0.0657 | 1.0608 | 0.9999 |
| NaY | 1.5242 | 0.0441 | 1.4077 | 1.3894 | 0.0437 | 0.6141 | 0.9999 |
| ZSM-5 | 1.6106 | 0.1899 | 0.7093 | 0.8785 | 0.1568 | 0.1310 | 0.9999 |

**Table S3.** The fitting parameters for single-site Langmuir-Freundlich (SSLF) isotherm model of $CF_4$ at 298 K.

| $CF_4$ | $q_{sat}$ | $b$ | $v$ | $R^2$ |
|---|---|---|---|---|
| Zn(BPZ) | 3.5429 | 0.0106 | 1.0178 | 0.9999 |
| Ni(BPZ) | 2.7356 | 0.0373 | 0.9400 | 0.9999 |
| Co(BPZ) | 2.8486 | 0.0113 | 0.9857 | 0.9999 |
| Cu(BPZ) | 1.2706 | 0.0316 | 0.8756 | 0.9999 |
| Zn(BPZ)-2Me | 2.7457 | 0.0137 | 0.9252 | 0.9999 |
| Zn(BDP) | 27.9610 | 0.0002 | 1.0495 | 0.9999 |
| Ni(BDP) | 8.4604 | 0.0012 | 1.0241 | 0.9999 |
| Fe(BDP) | 2.1748 | 0.0103 | 0.9994 | 0.9999 |
| NaY | 6.7297 | 0.0016 | 1.0415 | 0.9999 |
| ZSM-5 | 2.1208 | 0.0191 | 1.0100 | 0.9999 |

**Table S4.** Virial fitting parameters for $CF_4$ and $C_2F_6$ in Ni(BPZ) and Zn(BPZ).

| Parameters | $C_2F_6$ | | $CF_4$ | |
|---|---|---|---|---|
| | Zn(BPZ) | Ni(BPZ) | Zn(BPZ) | Ni(BPZ) |
| a0 | -3452.9432 | -4241.2565 | -2411.6381 | -3207.8699 |
| a1 | -1452.3136 | -3164.8916 | -13.7477 | -78.6050 |
| a2 | 1142.2153 | 3884.1231 | 281.9127 | 368.6307 |
| a3 | -605.1601 | -3088.2695 | -273.8596 | -175.1455 |
| a4 | 223.0417 | 1658.0085 | 113.0661 | 78.0002 |
| a5 | -25.9590 | -299.2973 | -16.4486 | -11.7219 |
| b0 | 11.6465 | 14.0445 | 11.4245 | 13.1662 |
| b1 | 4.0499 | 8.3863 | -0.1764 | 0.3570 |
| b2 | -1.4043 | -4.6773 | 0.1047 | -0.4771 |

**Table S5.** Summary of the separation performance for $CF_4$ and $C_2F_6$ in representative materials.

| Materials | $C_2F_6$ uptake at 3 kPa (mmol $g^{-1}$) | $C_2F_6$ uptake at 100 kPa (mmol $g^{-1}$) | $C_2F_6/CF_4$ selectivity (3/97) at 298 K and 100 kPa |
|---|---|---|---|
| Zn(BPZ) | 1.58 | 2.90 | 24.80 |
| Ni(BPZ) | 1.44 | 2.27 | 13.30 |
| NaY | 0.38 | 2.06 | 10.20 |
| 13X (303 K)[10] | 0.21 | 1.53 | 8.60 |
| ZSM-5 | 0.64 | 1.52 | 8.40 |
| Activated carbon (303 K)[10] | 0.24 | 1.38 | 6.30 |
| $Fe_2(BDP)_3$ | 0.21 | 1.40 | 3.00 |
| Zn(BDP) | 0.13 | 4.10 | 5.20 |
| Ni(BDP) | 0.31 | 2.58 | 9.10 |
| Zn(BPZ)-2Me | 0.80 | 1.88 | 12.20 |
| Co(BPZ) | 0.93 | 2.11 | 17.10 |
| Cu(BPZ) | 0.44 | 0.74 | 6.30 |

**Table S6.** The energy under different loadings of $C_2F_6$ in the supercell of $[Ni(BPZ)]_8$ (kJ per mol adsorbate).

| $C_2F_6$ loadings | Host-guest binding energy | Guest-guest binding energy |
|---|---|---|
| 1 | 67.31 | - |
| 2 | 68.27 | 2.27 |
| 3 | 68.65 | 1.88 |
| 4 | 69.01 | 2.61 |

**Table S7.** The energy under different loadings of $CF_4$ in the supercell of $[Ni(BPZ)]_8$ (kJ per mol adsorbate).

| $CF_4$ loadings | Host-guest binding energy | Guest-guest binding energy |
|---|---|---|
| 1 | 41.57 | - |
| 2 | 40.07 | 2.51 |
| 3 | 40.10 | 3.72 |
| 4 | 40.46 | 2.68 |

**Table S8.** The energy under different loadings of $C_2F_6$ in the supercell of $[Zn(BPZ)]_8$ (kJ per mol adsorbate).

| $C_2F_6$ loadings | Host-guest binding energy | Guest-guest binding energy |
|---|---|---|
| 1 | 46.28 | - |
| 2 | 46.29 | 4.24 |
| 3 | 45.91 | 5.02 |
| 4 | 45.74 | 5.87 |

**Table S9.** The energy under different loadings of $CF_4$ in the supercell of $[Zn(BPZ)]_8$ (kJ per mol adsorbate).

| $CF_4$ loadings | Host-guest binding energy | Guest-guest binding energy |
|---|---|---|
| 1 | 31.65 | - |
| 2 | 30.82 | 2.19 |
| 3 | 30.77 | 3.10 |
| 4 | 31.02 | 3.46 |

## References


[1] C. Pettinari, A. Tăbăcaru, I. Boldog, K. V. Domasevitch, S. Galli, N. Masciocchi, *Inorg. Chem.* **2012**, *51*, 5235–5245.
[2] S. Tu, L. Yu, D. Lin, Y. Chen, Y. Wu, X. Zhou, Z. Li, Q. Xia, *ACS Appl. Mater. Interfaces* **2022**, *14*, 4242–4250.
[3] Z. R. Herm, B. M. Wiers, J. A. Mason, J. M. van Baten, M. R. Hudson, P. Zajdel, C. M. Brown, N. Masciocchi, R. Krishna, J. R. Long, *Science* **2013**, *340*, 960–964.
[4] J. JagieŁŁo, L. Czepirski, *Chem. Eng. Sci.* **1989**, *44*, 797–801.
[5] A. L. Myers, J. M. Prausnitz, *AIChE J.* **1965**, *11*, 121–127.
[6] B. Delley, *J. Chem. Phys.* **1990**, *92*, 508–517.
[7] B. Delley, *J. Chem. Phys.* **2000**, *113*, 7756–7764.
[8] S. J. Clark, M. D. Segall, C. J. Pickard, P. J. Hasnip, M. I. J. Probert, K. Refson, M. C. Payne, *Z. Für Krist. - Cryst. Mater.* **2005**, *220*, 567–570.
[9] J.-R. Li, R. J. Kuppler, H.-C. Zhou, *Chem. Soc. Rev.* **2009**, *38*, 1477–1504.
[10] N.-G. Ahn, S.-W. Kang, B.-H. Min, S.-S. Suh, *J. Chem. Eng. Data* **2006**, *51*, 451–456.
